# Women’s costs for accessing comprehensive sexual and reproductive health services: findings from an observational study in Johannesburg, South Africa

**DOI:** 10.1186/s12978-019-0842-2

**Published:** 2019-12-16

**Authors:** Naomi Lince-Deroche, Kaitlyn M. Berry, Cheryl Hendrickson, Tembeka Sineke, Sharon Kgowedi, Masangu Mulongo

**Affiliations:** 1 Health Economics and Epidemiology Research Office, Department of Internal Medicine, School of Clinical Medicine, Faculty of Health Sciences, University of the Witwatersrand, 39 Empire Road, Parktown, Johannesburg, South Africa; 2 Department of Global Health, Boston University School of Public Health, 801 Massachusetts Ave. 3rd Floor, Boston, MA 02118 USA; 3 Right to Care, Helen Joseph Hospital, Perth Road, Westdene, Johannesburg, South Africa

**Keywords:** Contraception, Cervical cancer, Breast cancer, Menstruation, Fertility, Menopause, Gender-based violence, Economic, Affordability

## Abstract

**Background:**

Evaluating progress towards the Sustainable Development Goal of universal access to sexual and reproductive (SRH) services requires an understanding of the health needs of individuals and what constitutes access to services. We explored women’s costs of accessing SRH services in Johannesburg, South Africa and contextualized costs based on estimates of household income.

**Methods:**

We conducted an observational study of women aged 18–49 at a public HIV treatment site and two public primary health care facilities from June 2015 to August 2016. Interviews assessed women’s SRH needs (for contraception, fertility problems, menstrual problems, menopause symptoms, sexually transmitted infections (STI), experiences of intimate-partner violence (IPV), and cervical and breast cancer screening) and associated costs. We calculated average and total costs (including out-of-pocket spending, lost income, and estimated value of time spent) for women who incurred costs. We also estimated the total and average costs of meeting all SRH needs in a hypothetical “full needs met” year. Finally, we contextualize SRH spending against a measure of catastrophic expenditure (> 10% of household income).

**Results:**

Among the 385 women who participated, 94.8% had at least one SRH need in the prior 12 months; 79.7% incurred costs for accessing care. On average, women spent $28.34 on SRH needs during the prior year. Excluding one HIV-negative woman who spent 112% of her annual income on infertility treatment, HIV-positive women spent more on average annually for SRH care than HIV-negative women. Sixty percent of women reported at least one unmet SRH need. If all participants sought care for all reported needs, their average annual cost would rise to $52.65 per woman. Only two women reported catastrophic expenditure – for managing infertility.

**Conclusions:**

SRH needs are constants throughout women’s lives. Small annual costs can become large costs when considered cumulatively over time. As South Africa and other countries grapple with increasing access to SRH services under the rubric of universal access, it is important to remember that individuals incur costs despite free care at the point of service. Policies that address geographic proximity and service quality would be important for reducing costs and ensuring full access to SRH services.

**Plain English summary:**

Literature on women’s financial and economic costs for accessing comprehensive sexual and reproductive health care in low- and middle-income countries is extremely limited, and existing literature often overlooks out-of-pocket costs associated with travel, child care, and time spent accessing services. Using data from a survey of 385 women from a public HIV treatment site and two public primary health care facilities in Johannesburg, we found nearly all women reported at least on sexual and reproductive health need and more than 75% of women incurred costs related to those needs. Furthermore, more than half of women surveyed reported not accessing services for their sexual and reproductive health needs, suggesting a total annual cost of more than $50 USD, on average, to access services for all reported needs. While few women spent more than 10% of their total household income on sexual and reproductive health services in the prior year, needs are constant and costs incur throughout a woman’s life suggesting accessing services to meet these needs might still result in financial burden. As South Africa grapples with increasing access to sexual and reproductive health services under the rubric of universal access, it is important to remember that individuals incur costs despite free care at the point of service. Policies that address geographic proximity and service quality would be important for reducing costs and ensuring full access to services.

## Background

The Sustainable Development Goals, and the Millennium Development Goals before them, include the goal of universal access to sexual and reproductive health (SRH) services [1]. The World Health Organization has released guidance over time on the types of services to be included in comprehensive SRH care [2], including for HIV-positive women specifically [3]. Recently, a new definition, expanding the list of “essential” SRH and rights-related services, has been proposed [4]. Evaluating progress towards achieving universal access to SRH services requires both an understanding of individual needs/health problems and what constitutes access to services, which has been defined as physical accessibility, acceptability, and financial affordability [5–7].

In South Africa, which is home to 20% of all people with HIV worldwide [8], all health care services are currently offered through two parallel systems: public and private. The public system is financed through public taxation and serves roughly 80% of the population [9]. Primary health care services are offered freely to individuals who do not have private health insurance; services are also free for all pregnant and lactating women and children under age six [10]. In contrast, the private health system is financed through payments from individuals or via privately purchased health insurance, which often requires users to co-pay for services. In response to access problems in the public sector and equity concerns, South Africa’s national government has announced plans to merge the public and private systems into a National Health Insurance (NHI) scheme [11], and planning and piloting for the new system are currently underway.

A challenge to NHI planning is the dearth of information available on access patterns and expenditure by individuals seeking health care. Currently, although offered freely to most people, accessing health services in South Africa’s public sector is not without cost. Few studies have attempted to document women’s (or men’s) *comprehensive* SRH needs or access to services. In this study, we aimed to contribute to health systems planning by exploring women’s costs of accessing comprehensive SRH services in Johannesburg, South Africa and to contextualize women’s costs based on estimates of household income. We included a special focus on HIV-positive women’s needs and costs because of the high HIV prevalence in this setting, and the growing acknowledgement of HIV-positive women’s special needs regarding SRH care.

## Methods

We conducted an observational study at three public health facilities in Johannesburg, South Africa. One site, located within a large tertiary facility, offered comprehensive, outpatient HIV treatment and management services to HIV-positive individuals; the other two were primary health care facilities located close to the tertiary facility (2 and 7 km away) offering both HIV care and treatment and other primary health care services, including SRH services. The catchment area for the tertiary facility was broad. The facility is one of South Africa’s largest HIV treatment sites and has been operational for over a decade. The two primary health clinics had smaller catchment areas representing established urban areas.

### Data collection

From June 2015 to August 2016, trained interviewers used systematic random sampling to select and approach women in the waiting room queues of the three study facilities and assessed their interest in, and eligibility for, study participation. Women were eligible for inclusion in the study if they were: aged 18–49; able to speak English, Zulu, or Sotho; attending the clinic to obtain SRH, HIV or primary health care services for themselves (as opposed to their child or an accompanying friend, etc.); and willing to discuss HIV, including their own status if known. If interested and eligible, the women were asked to provide written consent and were interviewed in a private location at the study facility. The questions included demographics and socioeconomic characteristics, women’s self-reported SRH health needs – usually over the prior 12 months, whether the women had tried to access care for their needs, barriers to access, the costs associated with accessing care, perceptions of service quality, and preferences for service integration. Further detail on specific questions is provided below and the questionnaire is attached (see Additional File 1).

### Data management and analysis

Interview data were double-entered into REDCap [12], harmonized through data cleaning activities, and then exported to Stata (Release 15. College Station, TX: StataCorp LP) for analysis. For descriptive categorical data, we calculated proportions based on non-missing responses. For descriptive continuous variables, we calculated medians and interquartile ranges (IQRs) due to non-normal distribution of the data. Following STROBE guidelines for observational studies, we present demographic and socioeconomic information without statistical testing [13]. We present all results by HIV status (HIV-positive versus HIV-negative or status unknown).

We present women’s reported needs for SRH services over the past 12 months. For fertility problems, menstrual problems, menopause symptoms, sexually transmitted infections (STI), and experiences of intimate-partner violence (IPV), we determined need based on the women’s self-reported problems or symptoms. For contraception, we determined a woman to be in need of contraception if she was sexually active in the past year, not currently pregnant, not previously sterilized (for her or her partner), not wanting a child in next 24 months, and not menopausal. For breast and cervical cancer screening, we present whether the woman ever had a screen, and whether she needed a screen based on South Africa’s national policies for screening, which are dependent on age and HIV status (for cervical cancer screening) or age alone (for breast cancer screening).

All women who sought care for their needs in the last 12 months were asked for detailed information about where the care was sought. Some women visited more than one location to get care during the year. Our survey allowed for data collection on up to three service location types for each SRH service need. For this analysis, we collapsed the reported service location types into three categories: 1) public facilities, 2) private medical facilities/chemists/pharmacists, and 3) other, which included traditional healers, family members, friends, and other responses. Our survey also allowed women to report multiple visits to any of the three service locations during the year.

Women who sought care were also asked about costs associated with trying to obtain SRH services, including time and travel costs. We first calculated the mean cost and standard deviation for each health issue (e.g. fertility care, contraception, STIs, etc.) and cost type (e.g. out-of-pocket spending, lost income, and estimated value of time spent) considering only the women who incurred those costs. Then we summed out-of-pocket spending, lost income, and the value of time spent to estimate the average total costs incurred per women for each health issue. For formally and self-employed individuals who reported their monthly income, women’s time was valued using an approximated hourly wage calculated by dividing the reported monthly wage by 4.33 weeks per month and dividing that figure by 40 h per week. For unemployed and retired individuals, students with no income, and employed individuals who failed to report their monthly income, time was valued based on an hourly rate approximated using South Africa’s median monthly earnings estimate for women of 2833 Rand per month (approximately $193) in 2016 [14]. We also report whether anyone helped the women pay for their expenses (transport, fees, etc.) for each service category.

Next, we estimated the total annual cost to the study cohort for their reported accessing of all SRH services by summing the total costs reported for all women who accessed services of any kind. Dividing total costs by the number of women in the study cohort, we produced an average cost spent per woman for seeking SRH care during the prior 12 months.

Then, because some women had SRH needs for which they did not obtain care, we estimated the total and average costs of meeting all SRH needs for the study cohort in a hypothetical “full needs met” year. Need was again based on women’s reported needs—or our pre-defined estimates of need (i.e. for contraception and cervical and breast cancer screening). We estimated the total costs by adding women’s reported costs (for services where women managed to access SRH care) to estimated costs for accessing SRH services where women had a need for care but did not actually obtain the care. To estimate the costs of getting care for needs that were reportedly unaddressed, we assumed the mean costs per service from women who reported obtaining the service.

Finally, to contextualize women’s costs, we estimated average household income for the cohort. Women were asked to report monthly household receipt of grants from the South African government and average monthly income for themselves and their household. Where total household income was unknown by the women, we approximated total household income using the gender of the reported primary earner, or “breadwinner,” and national median monthly income figures for men (R 3738, or $254) and women (R 2833, or $193) in 2016 [14]. Due to a lack of data on overall household expenditure, we discuss the affordability of obtaining care for SRH needs in the context of a normative income guideline where costs exceeding 10% of overall household income are deemed catastrophic [15, 16].

All out-of-pocket costs, lost income, and reported and estimated individual and household income figures were collected in South African Rand (R). Costs were inflated to 2016 values (where necessary) using the Consumer Price Index reported in the International Monetary Fund’s World Economic Outlook database [17]. Lost income and individual and household income were inflated using World Bank Gross Domestic Product Deflators [18]. We present costs in 2016 US dollars (USD) using an average exchange rate for 2016 of 14.70236 Rands per dollar [19].

## Results

We approached 690 women at the three public health facilities during recruitment; 55.8% agreed to participate, resulting in a sample size of 385 women. Half of the women were recruited from the outpatient HIV clinic (*n* = 192, 49.9%), and the other half were recruited from the two primary health care facilities (*n* = 193, 50.1%). Among the 305 women who were approached but did not enrol, over two-thirds (*n* = 214, 70.2%) were not eligible (*n* = 152) or not interested to participate (*n* = 62). An additional 85 women (27.9% of those who did not enrol) were eligible but did not participate because they were in a hurry to leave the clinic.

The median age of the sample was 36.7 (IQR: 29.7–41.9), and HIV-negative women tended to be younger than HIV-positive women (Table 1). The women were predominately Black (87.5%), married (25.7%) or cohabitating (54.6%), and living in a house (42.9%). Most women reported completing Grade 8–11 (46.5%) or Grade 12/matriculated (32.0%). While over half the sample was employed (56.4%) and almost half reported being the primary breadwinner at home (48.1%), a majority of women (64.9%) reported that their primary source of income in the last 12 months was a transfer from a family member, spouse, or partner. The average monthly reported individual income was $262.31 while the average monthly reported household income was $438.95. After adjusting for cases where the total household income was unknown, the average monthly estimated household income for the cohort rose to $488.18.

**Table 1:** Demographics and socioeconomic status, *N* = 385 (n(%))

	HIV-negative/ status unknown	HIV-positive	Total
	(*n* = 129)	(*n* = 256)	(n = 385)
Age (median (IQR))	27.7 (22.1–37.7)	38.9 (34.0–42.6)	36.7 (29.7–41.9)
Race			
Black	94 (72.9%)	243 (94.9%)	337 (87.5%)
White	1 (0.8%)	1 (0.4%)	2 (0.5%)
Coloured^a^	34 (26.4%)	12 (4.7%)	46 (12.0%)
Indian/Other Asian	0 (0.0%)	0 (0.0%)	0 (0.0%)
Marital status			
Married (legal or informal)	38 (29.5%)	61 (23.8%)	99 (25.7%)
Cohabitating/has a partner	75 (58.1%)	135 (52.7%)	210 (54.6%)
Divorced/separated/widowed	3 (2.3%)	16 (6.3%)	19 (4.9%)
Single	13 (10.1%)	44 (17.2%)	57 (14.8%)
Education			
Grade 7 or less	8 (6.2%)	21 (8.2%)	29 (7.5%)
Grade 8–11	63 (48.8%)	116 (45.3%)	179 (46.5%)
Grade 12 / Matriculated	32 (24.8%)	91 (35.6%)	123 (32.0%)
Higher degree /diploma post matriculation	26 (20.2%)	28 (10.9%)	54 (14.0%)
Housing type			
House	62 (48.1%)	103 (40.2%)	165 (42.9%)
Flat/apartment	14 (10.9%)	39 (15.3%)	53 (13.8%)
Shack	20 (15.5%)	43 (16.8%)	63 (16.4%)
Wendy house/cottage/back room	15 (11.6%)	46 (18.0%)	61 (15.8%)
Domestic quarters/room in employers house	8 (6.2%)	19 (7.4%)	27 (7.0%)
Student residence	9 (7.0%)	3 (1.2%)	12 (3.1%)
Other	1 (0.8%)	3 (1.2%)	4 (1.0%)
Number of rooms in housing (median (IQR))	3 (1–4)	3 (1–4)	3 (1–4)
Electricity in housing	117 (90.7%)	230 (89.8%)	347 (90.1%)
Piped water in housing	92 (71.3%)	165 (64.5%)	257 (66.8%)
Number of people staying in housing (median (IQR))	4 (3–5)	3 (2–5)	4 (2–5)
Number of dependents (adults or children) (median (IQR))	1 (0–3)	3 (1–4)	2 (0–4)
Goes without food^b^			
Often	1 (0.8%)	2 (0.8%)	3 (0.8%)
Sometimes	31 (24.0%)	55 (21.5%)	86 (22.3%)
Seldom	1 (0.8%)	7 (2.7%)	8 (2.1%)
Never	96 (74.4%)	190 (74.2%)	286 (74.3%)
Employment			
Unemployed	60 (46.5%)	71 (27.7%)	131 (34.0%)
Employed (formal/informal)	55 (42.6%)	162 (63.3%)	217 (56.4%)
Self-employed (formal/informal)	4 (3.1%)	19 (7.4%)	23 (6.0%)
Unable to work/retired	1 (0.8%)	3 (1.2%)	4 (1.0%)
Student	9 (7.0%)	1 (0.4%)	10 (2.6%)
She is primary breadwinner at home	33 (25.6%)	152 (59.4%)	185 (48.1%)
If not, primary breadwinner is male	68 (70.8%)	80 (76.9%)	148 (74.0%)
Primary source of income in the last 12 months			
None	13 (10.1%)	9 (3.5%)	22 (5.7%)
Employment/working (formal or informal)	29 (22.5%)	31 (12.1%)	60 (15.6%)
Transfer from family member, spouse, partner	63 (48.8%)	187 (73.1%)	250 (64.9%)
Grant	23 (17.8%)	25 (9.8%)	48 (12.5%)
Rental income from tenants	1 (0.8%)	4 (1.6%)	5 (1.3%)
Monthly reported individual income (mean (SD))	$265.93 ($944.47)	$260.35 ($273.11)	$262.31 ($600.31)
Monthly reported household income (including respondent) (mean (SD))	$496.89 ($1167.93)	$409.52 ($498.29)	$438.95 ($789.45)
Monthly *estimated* household income (mean (SD))^c^	$568.19 ($1146.06)	$447.87 ($473.63)	$448.18 ($768.12)

*PHC* Primary health clinic, *IQR* interquartile range

^a^ “Coloured” is an official ethnic category in South Africa used to denote individuals of mixed race

^b^Missing = 2

^c^Estimated household income includes approximation of “breadwinner” income if unknown by the respondent

### SRH service needs and access patterns

Among the 385 women who participated in the study, 94.8% had at least one SRH need during the prior 12 months (Table 2). The most commonly reported SRH service needs were contraception (45.7%) and care for self-reported menstrual problems (39.2%). Reports of menopause symptoms (15.9%), STI symptoms (13.9%), and IPV (10.9%) were comparatively less common. Over a third of all women who tried to become pregnant in the last year (*n* = 40) reported experiencing fertility problems (*n* = 15, 37.5%). Based on South Africa’s policies for cervical and breast cancer screening, 81.8 and 58.7% of women were eligible for a screen at the time of their interview based on age and/or HIV status.

**Table 2:** Women’s self-reported sexual and reproductive health care needs and service access patterns in the last 12 months, N = 385 (n(%))

	HIV-negative/ status unknown	HIV-positive	Total
	(*n* = 129)	(*n* = 256)	(*n* = 385)
Required care for at least one SRH problem	109 (84.50%)	256 (100.0%)	365 (94.8%)
Required care for fertility problems	4 (3.1%)	11 (4.3%)	15 (3.9%)
Sought care^a^	4 (100.0%)	9 (81.8%)	13 (86.7%)
Public facility	4 (100.0%)	5 (55.6%)	9 (69.2%)
Private facility or chemist	0 (0.0%)	1 (11.1%)	1 (7.7%)
Other^b^	1 (25.0%)	4 (44.4%)	5 (38.5%)
Needed contraception^c^	63 (48.8%)	113 (44.1%)	176 (45.7%)
Currently using a modern form of contraception	56 (88.9%)	107 (94.7%)	163 (92.6%)
Sought contraception in last 12 months*	48 (76.2%)	89 (78.8%)	137 (77.8%)
Public facility	44 (91.7%)	60 (67.4%)	104 (75.9%)
Private facility or chemist	2 (4.2%)	15 (16.9%)	17 (12.4%)
Other^b^	2 (4.2%)	14 (15.7%)	16 (11.7%)
Reported having menstrual problems	48 (37.2%)	103 (40.2%)	151 (39.2%)
Sought care^a^	25 (52.1%)	49 (47.6%)	74 (49.0%)
Public facility	21 (84.0%)	37 (75.5%)	58 (78.4%)
Private facility or chemist	2 (8.0%)	9 (18.4%)	11 (14.9%)
Other^b^	2 (8.0%)	6 (12.2%)	8 (10.8%)
Reported having menopause symptoms	12 (9.3%)	49 (19.3%)	61 (15.9%)
Sought care^a^	5 (41.7%)	17 (34.7%)	22 (36.1%)
Public facility	3 (60.0%)	14 (82.4%)	17 (77.3%)
Private facility or chemist	2 (40.0%)	2 (11.8%)	4 (18.2%)
Other^b^	1 (20.0%)	1 (5.9%)	2 (9.1%)
Eligible for a cervical cancer screen^d^	59 (45.7%)	256 (100%)	315 (81.8%)
Ever had a cervical cancer screen	44 (74.6%)	211 (82.8%)	255 (81.2%)
Sought a cervical cancer screen in last 12 months	10 (22.7%)	106 (50.2%)	116 (45.5%)
Public facility	15 (88.2%)	105 (99.1%)	120 (97.6%)
Private facility or chemist	2 (11.8%)	0 (0.0%)	2 (1.6%)
Other^b^	0 (0.0%)	1 (0.9%)	1 (0.8%)
Eligible for a breast cancer screen in last 12 months^e^	45 (34.9%)	181 (70.7%)	226 (58.7%)
Ever had a breast cancer screen	9 (20.0%)	32 (17.7%)	41 (18.1%)
Sought a breast cancer screen in last 12 months^a^	1 (11.1%)	12 (37.5%)	13 (31.7%)
Public facility	6 (85.7%)	17 (94.4%)	23 (92.0%)
Private facility or chemist	1 (14.3%)	1 (5.6%)	2 (8.0%)
Other^b^	0 (0.0%)	0 (0.0%)	0 (0.0%)
Reported having STI symptoms	16 (12.4%)	37 (14.6%)	53 (13.9%)
Sought care^a^	15 (93.8%)	28 (75.7%)	43 (81.1%)
Public facility	9 (60.0%)	24 (85.7%)	33 (76.7%)
Private facility or chemist	3 (20.0%)	3 (10.7%)	6 (14.0%)
Other^b^	3 (20.0%)	3 (10.7%)	6 (14.0%)
Reported experiencing intimate partner violence	11 (8.5%)	31 (12.1%)	42 (10.9%)
Sought care^a^	4 (36.4%)	8 (25.8%)	12 (28.6%)
Public facility	1 (25.0%)	1 (12.5%)	2 (16.7%)
Private facility or chemist	0 (0.0%)	0 (0.0%)	0 (0.0%)
Other^b^	0 (0.0%)	2 (25.0%)	2 (16.7%)

*STI* Sexually transmitted illness

^a^Women may have obtained care at more than one facility or mode (e.g. public facility, traditional healer, etc)

^b^Other = traditional healer, family member, friend, other

^c^ Need for contraception was defined as having had sex in the last 12 months, not currently pregnant, not having had tubal ligation or a partner with a vasectomy, not wanting to get pregnant in the next 24 months and not experiencing menopause symptoms

^d^ In South Africa, HIV-negative women are eligible for Pap smears at ages 30, 40, and 50. For this analysis, we considered any HIV-negative woman over age 30 to be eligible. HIV-positive women are eligible for annual Pap smears from the time of their diagnosis

^e^ Breast exams in South Africa are clinical exams. The national policy allows for a screen annually for women over age 35

Care seeking behaviour, defined as the percentage of women who reported seeking care among those who needed care, differed by type of service. Roughly four out of five women who experienced fertility problems, needed contraception, or had STI symptoms sought care for their need. In contrast, less than half of women with a need for care related to menstrual problems, menopause symptoms, or experiences of IPV sought care. The majority of eligible women reported ever having a cervical cancer screen (81.2%), while only 18.1% of eligible women had ever had a breast cancer screen. Reports of ever having a cervical cancer screen were more common among women who were HIV-positive than women who were HIV-negative or who did not know their status.

Considering where women accessed care, many women (67.1%) sought care in public facilities. However, seeking care in private facilities or from private chemists also occurred. Among women who sought care for at least one SRH need, 10.7% sought care in a private facility or chemist.

### Reported costs and time spent accessing SRH services

For each type of SRH service, women who sought care reported, on average, between one and three visits to any facility in the last 12 months (Table 3). Considering women’s combined SRH needs, women made an average of 2.2 visits to facilities annually.

**Table 3:** Among women who received care, women’s costs (2016 USD) (mean (SD)) and assistance for obtaining sexual and reproductive health services in the last 12 months, *N* = 385

	HIV-negative/ status unknown	HIV-positive	Total
	(*n* = 129)	(*n* = 256)	(*n* = 385)
Incurred costs for at least one SRH problem	98 (76.0%)	209 (81.6%)	307 (79.7%)
Had fertility problems and tried to seek help	*n* = 4	*n* = 9	*n* = 13
Number of visits to facilities	5.8 (3.9)	1.6 (1.1)	2.8 (2.9)
If employed and sought care, missed work?	1 of 2 (50.0%)	3 of 8 (37.5%)	4 of 10 (40.0%)
If yes, number of days	10 (0)	1 (0)	3.3 (4.5)
If yes, lost income	–	$15.62 ($8.73)	$15.62 ($8.73)
If spent time travelling or waiting, average hours spent	18.4 (16.2)	4.1 (3.2)	8.5 (10.9)
Value of time spent	$18.56 ($16.65)	$4.20 ($2.73)	$8.62 ($11.04)
Average out-of-pocket costs, if incurred	$927.30 ($1294.38)	$53.53 ($65.03)	$228.28 ($570.24)
Average total costs for all women with issue^a^	$482.21 ($924.43)	$55.25 ($70.81)	$186.62 ($508.97)
Received financial help to obtain care (n (%))	0 (0.0%)	3 (33.3%)	3 (23.1%)
Sought contraception in last 12 months	*n* = 48	*n* = 89	*n* = 137
Number of visits to facilities	1.3 (0.8)	1.7 (1.6)	1.5 (1.4)
If employed and sought care, missed work?	5 of 22 (22.7%)	9 of 57 (15.8%)	14 of 79 (17.7%)
If yes, number of days	1.2 (0.4)	1 (0.0)	1.1 (0.3)
If yes, lost income	$7.48 ($1.84)	$26.01 ($13.40)	$16.75 ($13.29)
If spent time travelling or waiting, average hours spent	3.3 (3.8)	3.9 (10.2)	3.6 (8.3)
Value of time spent	$3.21 ($2.62)	$5.51 ($16.18)	$4.65 ($12.91)
Average out-of-pocket costs, if incurred	$8.38 ($14.67)	$10.73 ($17.48)	$10.14 ($16.72)
Average total costs for all women with issue^a^	$6.27 ($9.29)	$12.44 ($28.13)	$10.13 ($23.09)
Received financial help to obtain care (n (%))	4 (8.3%)	13 (14.6%)	17 (12.4%)
Sought care for menstrual problems	*n* = 25	*n* = 49	*n* = 74
Number of visits to facilities	1.9 (1.7)	2.3 (1.6)	2.1 (1.6)
If employed and sought care, missed work?	7 of 18 (38.9%)	11 of 27 (40.7%)	18 of 45 (40.0%)
If yes, number of days	1.7 (1.0)	1.5 (1.3)	1.6 (1.1)
If yes, lost income	$16.19 ($13.17)	$18.78 ($20.09)	$17.48 ($16.07)
If spent time travelling or waiting, average hours spent	6.9 (9.4)	5.9 (7.1)	6.2 (7.9)
Value of time spent	$7.45 ($10.53)	$8.22 ($10.83)	$7.95 ($10.66)
Average out-of-pocket costs, if incurred	$56.78 ($85.28)	$31.62 ($51.68)	$36.76 ($59.75)
Average total costs for all women with issue^a^	$31.13 ($63.17)	$33.59 ($52.62)	$32.74 ($56.07)
Received financial help to obtain care (n (%))	4 (16.0%)	9 (18.4%)	13 (17.6%)
Sought care for menopause symptoms	*n* = 5	*n* = 17	*n* = 22
Number of visits to facilities	3.0 (3.5)	1.4 (0.8)	1.7 (1.8)
If employed and sought care, missed work?	0 of 3 (0.0%)	3 of 8 (37.5%)	3 of 11 (27.3%)
If yes, number of days	–	1.7 (1.2)	1.7 (1.2)
If yes, lost income	–	$19.61 ($14.58)	$19.61 ($14.58)
If spent time travelling or waiting, average hours spent	14.2 (28.1)	7.2 (11.7)	8.8 (16.2)
Value of time spent	$15.02 ($23.50)	$7.90 ($12.16)	$9.52 ($15.13)
Average out-of-pocket costs, if incurred	$28.25 ($21.81)	$21.60 ($36.10)	$22.77 ($33.54)
Average total costs for all women with issue^a^	$31.97 ($26.71)	$29.14 ($38.69)	$29.77 ($35.74)
Received financial help to obtain care (n (%))	0 (0.0%)	5 (29.4%)	5 (22.7%)
Sought a cervical cancer screen	*n* = 17	*n* = 106	*n* = 123
Number of visits to facilities	1 (0.0)	1.2 (0.5)	1.1 (0.4)
If employed and sought care, missed work?	2 of 10 (20.0%)	27 of 76 (35.5%)	29 of 86 (33.7%)
If yes, number of days	1 (0.0)	1.0 (0.2)	1.0 (0.2)
If yes, lost income	$10.20 ($0.00)	$21.60 ($34.79)	$20.72 ($33.45)
If spent time travelling or waiting, average hours spent	2.9 (2.0)	2.1 (1.6)	2.2 (1.7)
Value of time spent	$3.89 ($2.81)	$3.09 ($4.29)	$3.20 ($4.11)
Average out-of-pocket costs, if incurred	$84.14 ($128.40)	$4.84 ($6.38)	$8.81 ($31.10)
Average total costs for all women with issue^a^	$24.29 ($66.58)	$9.06 ($15.76)	$11.19 ($28.77)
Received financial help to obtain care (n (%))	1 (5.9%)	14 (13.2%)	15 (12.2%)
Sought a breast cancer screen	*n* = 7	*n* = 18	*n* = 25
Number of visits to facilities	1.1 (0.4)	1.4 (1.2)	1.4 (1.0)
If employed and sought care, missed work?	1 of 3 (33.3%)	5 of 13 (38.5%)	6 of 16 (37.5%)
If yes, number of days	2 (0.0)	1 (0.0)	1.3 (0.5)
If yes, lost income	$12.24 ($0.00)	$65.37 ($92.45)	$47.66 ($72.21)
If spent time travelling or waiting, average hours spent	2.5 (2.2)	2.6 (2.6)	2.6 (2.4)
Value of time spent	$2.85 ($2.43)	$5.58 ($9.29)	$4.81 ($8.01)
Average out-of-pocket costs, if incurred	$1.09 ($0.19)	$6.72 ($7.44)	$5.91 ($7.14)
Average total costs for all women with issue^a^	$4.91 ($4.46)	$17.32 ($34.11)	$13.85 ($29.35)
Received financial help to obtain care (n (%))	1 (14.3%)	2 (11.1%)	3 (12.0%)
Sought care for an STI	*n* = 15	*n* = 28	*n* = 43
Number of visits to facilities	1.1 (0.3)	1.9 (1.5)	1.6 (1.3)
If employed and sought care, missed work?	4 of 7 (57.1%)	11 of 21 (52.4%)	15 of 28 (53.6%)
If yes, number of days	1.3 (0.5)	2.7 (4.0)	2.3 (3.4)
If yes, lost income	$13.60 ($6.80)	$26.22 ($28.91)	$22.01 ($23.95)
If spent time travelling or waiting, average hours spent	2.3 (1.4)	5.1 (6.4)	4.1 (5.3)
Value of time spent	$2.12 ($1.50)	$6.97 ($11.19)	$5.27 ($9.31)
Average out-of-pocket costs, if incurred	$18.67 ($16.36)	$23.12 ($32.81)	$21.82 ($28.69)
Average total costs for all women with issue^a^	$13.55 ($17.45)	$26.62 ($42.22)	$22.06 ($35.88)
Received financial help to obtain care (n (%))	2 (13.3%)	4 (14.3%)	6 (14.0%)
Sought treatment/care for gender-based violence	*n* = 4	*n* = 8	*n* = 12
Number of visits to facilities	3.5 (4.4)	2.9 (1.7)	3.1 (2.7)
If employed and sought care, missed work?	1 of 1 (100.0%)	2 of 5 (40.0%)	3 of 6 (50.0%)
If yes, number of days	2.0 (0.0)	1.7 (1.2)	1.8 (1.0)
If yes, lost income	$12.24 ($0.00)	$108.96 ($0.00)	$60.60 ($68.39)
If spent time travelling or waiting, average hours spent	5.0 (6.7)	6.2 (7.2)	5.8 (6.7)
Value of time spent	$5.36 ($7.60)	$36.13 ($84.07)	$24.94 ($67.08)
Average out-of-pocket costs, if incurred	$4.15 ($0.00)	$21.49 ($17.63)	$18.02 ($17.13)
Average total costs for all women with issue*	$9.46 ($6.93)	$63.97 ($100.99)	$44.15 ($83.00)
Received financial help to obtain care (n (%))	1 (25.0%)	0 (0.0%)	1 (8.3%)

^a^Total costs = out-of-pocket expenditure + value of time spent + lost income for all visits for the health problem

Among women who were employed, many missed work while trying to obtain needed SRH care. For STI symptoms 53.6% of those who were employed and sought care missed work; for IPV it was 50.0%; menstrual problems 40.0%; and fertility problems 40.0%. In contrast, only 17.7% of employed women who sought contraception had to miss work to obtain it.

Time spent seeking care over the last 12 months varied greatly both within and across service types. For example, individual women reported a wide range of time spent traveling and waiting for care for one health problem, and total time spent varied, on average, from just over two hours for cervical cancer screening to more than eight hours for fertility problems or menopause symptoms.

Out-of-pocket costs included spending on travel or other items or services associated with seeking SRH care over the last 12 months. Many women who sought care incurred out-of-pocket costs. Among women who sought care for any SRH need, 43.1% spent money on travel, 8.8% spent on consultations, 19.0% spent on medications, and 9.9% spent on childcare or other items. Women’s total out-of-pocket spending varied between the types of services. The average out-of-pocket cost was less than $25 for women who sought care related to contraception, menopause symptoms, cervical cancer screening, breast cancer screening, STI symptoms, and IPV, while women seeking care for fertility problems and menstrual problems reported spending more.

Considering a 12-month period, the average total costs, which included out-of-pocket expenditure plus the value of time spent and reported lost income, were under $50 for seeking care related to contraception, menstrual problems, menopause symptoms, cervical cancer screening, breast cancer screening, STI symptoms, and IPV. The average total cost for seeking care for fertility problems, however, was $186.62 though this was driven largely by a single woman who reported spending $1868.48 on fertility care.

Among all women enrolled in the study, 307 women (79.7%) reportedly incurred costs for at least one SRH need in the prior 12 months. Many (83.1%) had more than one SRH need. However, looking across all types of SRH services, only a minority of women reported that anyone helped them pay for the expenses incurred in obtaining SRH care.

### Women’s total annual costs for meeting all SRH needs and affordability

The total annual cost to the study cohort for seeking care for SRH needs, based on women’s reported care seeking behaviour, was $10,911.77, or an average cost of $28.34 per woman (Table 4). The average annual cost per HIV-negative or status-unknown woman for managing her SRH needs was higher ($32.84) than the average cost per HIV-positive woman ($26.07). However, the average annual cost for HIV-negative women was skewed by spending by one woman. When that outlier was removed from the data, the average annual cost for HIV-negative women dropped to $18.22. Further, the median cost of managing SRH needs among all HIV-negative women was $4.32 as compared to $7.00 among all HIV-positive women. Women aged 35 years or old also had higher than average costs ($37.45 per woman). Older women had higher costs than younger women in all SRH categories, with the biggest difference in spending on care related to fertility problems and intimate partner violence.

**Table 4:** Total estimated costs (2016 USD) per cohort and per woman for accessing needed sexual and reproductive health services over 12 months, *N* = 385

	2016 USD
Total annual cost to study cohort for accessing services *as reported*	$10,911.77
Cost per woman (mean (SD))	$28.34 ($113.95)
HIV-positive	$26.07 ($54.66)
HIV-negative/status unknown^a^	$32.84 ($181.58)
Women aged 35 or older	$37.45 ($145.17)
Total annual cost to study cohort for accessing services *to meet all needs*	$18,912.95
Cost per woman (mean (SD))	$52.65 ($118.63)
HIV-positive	$52.38 ($64.83)
HIV-negative/status unknown^b^	$53.20 ($183.97)
Women aged 35 or older	$62.80 ($148.43)

^a^The average annual cost for HIV-negative women was skewed by spending by one woman. When that outlier was removed from the data, the average annual cost for HIV-negative women dropped to $18.22 ($46.11)

^b^Removing the outlier, the average annual cost for HIV-negative women to meet all needs dropped to $38.25 ($53.33)

Additionally, some women had unmet SRH needs, or SRH needs for which they did not seek care. Using estimated costs for meeting those unmet SRH needs, we estimated that the total cost to the study cohort would have been $18,912.95 if women sought care for all reported SRH needs in the prior 12 months, resulting in an average cost of $52.65 per woman. While we found no difference in the average cost per HIV-positive woman versus a woman with negative or unknown status, we did find that women aged 35 or older would have higher costs to meet all reported needs compared to the overall cohort ($62.80 per woman).

Based on an average household income of between $438.95 and $488.18 per month, total spending as reported by women represented on average 0.9% (range 0–113%) of annual household income. To meet all SRH needs, spending would rise slightly to an average of 1.9% (range 0–114%) of annual household income. Only two women in the study cohort reported catastrophic spending (greater than 10% of annual income) on SRH services. These two women spent 13.9 and 112.2% of their total annual household income seeking care for SRH services over the prior 12 months. For these women, the cost drivers were intimate partner violence and fertility problems respectively. An additional three women had non-zero healthcare costs but reported $0 in annual household income.

## Discussion

SRH needs are commonplace for women throughout their lifespans. Among women in this study, who were aged 18–49, 94.8% had at least one SRH need in the past 12 months. Contraception was the most common SRH need among the cohort of women. However, other SRH needs not currently addressed by national level health policies in the country, including menstrual problems and menopause symptoms, were also prevalent.

Our data illustrate women’s access patterns when trying to address their SRH needs and point to possible gaps or successes in public sector service provision. Although the women were recruited in public health facilities, of those who sought care for an SRH need in the past year, one in ten sought care from a private facility or chemist. This may have been due to perceived access problems in the public sector or preferences for private care. Mixed use of the country’s public and private systems, including by individuals with private health insurance, has been reported previously [20, 21].

Based on South Africa’s policies for cervical and breast cancer screening, 81.8 and 58.7% of women were eligible for a screen at the time of their interview based on age and/or HIV status; and 81.2 and 18.1% of eligible women respectively had ever had a cervical or breast cancer screen. The high proportion of women with a cervical cancer screen may seem like a success, but in our study, it was largely due to half the study cohort being drawn from the outpatient HIV treatment facility. The facility has been supported for many years by a local NGO that provides cervical cancer screening and treatment on site. A study looking at screening rates among HIV-positive women nationwide estimated that only 26–41% of women obtain the recommended three-yearly screening [22]. Breast cancer screening is also lower than desired by policy makers in South Africa and contributes to poor health outcomes [23]. In this study, prior breast screening was uncommon despite, in theory, easy access given that there was a comprehensive breast care unit co-located within the tertiary facility (where the HIV treatment site was located, so just 2–7 km from the primary health facilities). The clinic provides a range of services from screening to advanced surgeries [24].

Despite primary health care services being offered freely in South Africa’s public health system, many women in this study incurred costs when accessing care for their SRH needs throughout the previous year: 79.7% incurred costs of any kind for addressing their SRH needs. This was not surprising in that, worldwide, individuals spend money on travel and miss work to obtain health care. However, in this study, many of the participants were unemployed and depending on transfers from family and friends as their primary source of monthly income.

In 2011, South Africans spent on average 1.4% of their household income (estimated to be R1,357, or roughly $186.91, per year) on health related expenditures [25]. That excluded spending on health insurance and transportation, which may have been for health-related reasons [25], so full costs may have been higher. We showed that to fully meet women’s annual SRH needs, including transportation to/from services, losing income and paying out-of-pocket costs, they would need to spend an average of 1.9% (range 0–114%) of household income.

This level of household spending may seem “affordable,” especially since few women experienced catastrophic levels of spending for their care. It is important to note, however, that the simple definition of catastrophic expenditure used in this analysis and widely used elsewhere (i.e. costs exceeding 10% of overall household income) is not without criticism. While using this objective definition allows for comparisons across settings or services, it does not fully acknowledge ability-to-pay [21]. It also fails to convey the impact of repeated expenditures – even if below the level of catastrophic expenditure. Thus, given that women’s SRH-related costs are likely to persist year-on-year and that so few women seem to receive help in paying for these costs, over the course of a woman’s life, the total costs could become substantial. Indeed, many women in our study did not obtain care for their SRH needs. This may reflect structural barriers to accessing care, but likely also reflects financial barriers faced by the women.

Finally, although mean annual expenditure for SRH needs was higher among HIV-negative women, it was largely driven by one woman who spent 112% of her annual household income on infertility treatment. When treated as an outlier and removed from the analysis, HIV-positive women spent more on average per year for SRH care. This finding highlights not only that infertility treatment (which is not available freely in South Africa’s public sector) is hugely expensive for some women, but also that HIV-positive women may need special attention in terms of assistance with accessing SRH services.

This study has limitations. The women were recruited from within public health care facilities and are likely not representative of all women aged 18–49 in Johannesburg in terms of their health care needs or ability to obtain care when needed. However, we feel that the costs incurred for obtaining care are likely generalizable to an urban population dependent on public sector health care. Some women in the study were not able to provide estimates of their total household income. They reported not knowing the income of others in their household, including in some cases, their spouse’s income. Our approach for valuing the income of households for cases where respondents reported not knowing the income follows generally accepted conventions, but is still an estimate at best. Finally, our approach for assessing affordability is crude. The normative 10%-of-household-income threshold has been criticized for its lack of nuance [21]; however, without better data on overall household spending, this crude measure may still provide insight as to the burden of health-related expenditure.

Literature on women’s financial and economic costs for accessing comprehensive SRH care in low- and middle-income countries is extremely limited. There are published accounts of women’s costs for accessing safe abortion in the public sector in South Africa [26, 27] and safe abortion and post-abortion care services in public and private facilities in Nigeria [28]. There are older and more recent accounts of the costs of *providing* family planning in developing countries [29–32]; however, these do not address women’s costs associated with accessing services. The recent Global Commodity Gap Analysis report highlights women’s out-of-pocket spending on contraception in the private sector, but not in public sector facilities [33]. There are also accounts in the literature on how affordability is or is not a barrier to accessing family planning services in low- and middle-income countries, though none that we could find empirically assessed women’s full costs and affordability.

## Conclusions

Access to health care is a complex concept. It includes physical accessibility, financial affordability, and acceptability [5–7]. Universal access implies that individuals are able to get quality care when needed, without financial risk [6]. Our study shows that most women incur costs for managing their SRH needs on an annual basis, and yet, among the study respondents, many were not able to access care for all of their needs. Actual spending on SRH needs, for those who accessed care, did not reach catastrophic levels (using a crude measure) for almost all women in the study. However, one must remember that SRH needs are constants throughout women’s lives, meaning that small costs can become large costs when considered cumulatively over time.

Ensuring universal access to health care implies that individuals recognize and understand their health care needs and that affordable services are available to them, offered by a health system that anticipates and can cater for the needs of its constituents. As South Africa and other countries grapple with plans for increasing access to SRH services under the rubric of universal access, it is important to remember that individuals incur costs despite free care at the point of service. Policies that address geographic proximity, access outside of working hours, and service quality would be important for reducing costs and ensuring full access to services.

## Supplementary information

**Additional file 1.** Semi Structured Interview (PDF) - Survey questionnaire

## Data Availability

The study data are available from the Health Economics and Epidemiology Research Office, but restrictions apply. Prior approval would need to be obtained from the Human Research Ethics Committee at the University of the Witwatersrand.

## Abbreviations

IPV: Intimate partner violence
SRH: Sexual and reproductive health
STIs: Sexually transmitted infections
