# Supplementary material for: Women’s costs for accessing comprehensive sexual and reproductive health services: findings from an observational study in Johannesburg, South Africa
Source: Reprod Health. 2019 Dec 16;16:179. doi: 10.1186/s12978-019-0842-2 (PMC6916226; doi:10.1186/s12978-019-0842-2)
Supplement: Supplementary file 1 — Additional file 1. Semi Structured Interview (PDF) - Survey questionnaire [file 12978_2019_842_MOESM1_ESM.pdf]

Today's Date					
d	d	m	m	y	y

Study ID				

Table of contents

Section	Sub-section	Page number
**Part A - Demographics and household income status**		2
**Part B – Health conditions, service quality**		6
**Part C – Sexual and Reproductive Health Needs Assessment and Costs**		7
	Fertility needs and costs	8
	Contraceptive needs	13
	Contraceptive costs	16
	Menstruation needs and costs	20
	Menopause needs and costs	24
	Cervical cancer needs and costs	29
	Breast care needs and costs	33
	STI needs and costs	38
	TOP needs and costs	42
	GBV needs and costs	46
**Part D – Service quality / integration**		52

Do Not Circulate

Today's Date					
d	d	m	m	y	y

Study ID				

i. **Facility ID**

Instructions: (Study staff) Read bold text aloud. Do not read ALL CAPS. For multi-choice questions, tick the appropriate box(es). For open responses, write the response in space provided.

Part A: Demographics and household income/status

Question	Response
**I'm going to start our interview by asking you some questions about your background and your household.**	
**1. What is your race?**	☐ 1 = Black African<br>☐ 2 = White<br>☐ 3 = Coloured<br>☐ 4 = Indian<br>☐ 5 = Other Asian<br>☐ 6 = Other
**2. Do you have a South African ID or are you a South African citizen?**	☐ 0= No<br>☐ 1= Yes (either ID or citizen)
**3. What is your marital status?**	☐ 1 = Married (legal or traditional)<br>☐ 2 = Have a partner, but not living together<br>☐ 3 = Cohabiting<br>☐ 4 = Divorced/separated<br>☐ 5 = Single<br>☐ 6 = Widowed
**4. What is the highest level of education that you have completed?** PROBE AS NECESSARY.	☐ 1 = Grade 5 or less<br>☐ 2 = Grade 6-7<br>☐ 3 = Grade 8-11<br>☐ 4 = Grade 12 / Matriculated<br>☐ 5 = Diploma/Certificate<br>☐ 6 = Degree (BA, Bcom, etc)<br>☐ 7 = Honours<br>☐ 8 = Masters<br>☐ 9 = Doctoral

Do Not Circulate

Today's Date					
d	d	m	m	y	y

Study ID				

Do Not Circulate

**5. What neighborhood do you stay in?**	List:<br><br>CODE:
**6. What type of housing do you live in?** PROBE AS NEEDED.	☐ 1 = House<br>☐ 2 = Flat/apartment<br>☐ 3 = Shack<br>☐ 4 = Wendy house/cottage/ back room (behind another house)<br>☐ 5 = Domestic quarters or room in employer's home<br>☐ 6 = Student residence<br>☐ 7 = Other SPECIFY: ___________
**7. How many rooms are there where you currently stay?** COUNT ALL SEPARATE ROOMS – E.G. KITCHEN, BEDROOMS, ETC. EXCLUDE BATHROOMS AND HALLS, PASSAGES.	Number: ______
**8. Do you have electricity in the place where you stay?**	❑ 0= No<br>❑ 1= Yes
**9. Do you have piped water inside the place where you stay?**	❑ 0= No<br>❑ 1= Yes
**10. How many adults and children including yourself have stayed at your place at least 15 out of the last 30 days?**	a) Adults: ______________<br>b) Children: ___________<br>Total: _______________
**11. How would you describe your employment situation?** PROBE AS NEEDED.	❑ 1= Unemployed looking for work<br>❑ 2 = Unemployed not looking for work<br>❑ 3 = Self-employed, formal or informal<br>❑ 4 = Employed, part-time or full-time<br>❑ 5 = Unable to work<br>❑ 6 = Retired<br>❑ 7 = Other *SPECIFY*: ______________________________

Today's Date					
d	d	m	m	y	y

Study ID				

**12. a. Are you the primary "breadwinner" in your household?** (IF MORE THAN ONE PERSON IS WORKING, THE QUESTION IS ASKING IF SHE EARNS THE MOST.)	❑ 0 = No<br>❑ 1 = Yes → *GO TO Q13*
b. IF 12a = NO, **Is the breadwinner a man or woman?**	❑ 1 = Man<br>❑ 2 = Woman
**c.** IF 12a = NO**, How would you describe the employment situation of the primary breadwinner in your household?** PROBE AS NEEDED.	❑ 1= Unemployed looking for work<br>❑ 2 = Unemployed not looking for work<br>❑ 3 = Self-employed, formal or informal<br>❑ 4 = Employed, part-time or full-time<br>❑ 5 = Unable to work<br>❑ 6 = Retired<br>❑ 7 = Other *SPECIFY*: ________________________
**13. a. Are you currently caring financially for any dependents (children or adults or both)?**	❑ 0 = No → GO TO Q14<br>❑ 1 = Yes
b. IF YES: **How many children, adults, or both are you taking care of financially?**	a) Children: _______<br>b) Adults: ________<br>c) Total: _________
**14. Do the people in your household go without food often, sometimes, seldom, or never?**	❑ 1 = Often<br>❑ 2 = Sometimes<br>❑ 3 = Seldom<br>❑ 4 = Never
**15. What has been your <u>primary</u> source of income (or money) in the last 12 months?** CHOOSE ONE ONLY.	❑ 0 = None<br>❑ 1 = Money given by family member, spouse, boyfriend /girlfriend<br>❑ 2 = Employment/working (formal or informal)<br>❑ 3 = Grant *(OWN GRANT ONLY, INCLUDES GRANT FOR HER CHILD)*<br>❑ 4 = Other *SPECIFY*: __________

Do Not Circulate

Today's Date					
d	d	m	m	y	y

Study ID				

**16. a. I'd like to know your average monthly income from work, grants or other sources. This will help us to understand whether getting health care services is sometimes a burden for you. Can you tell me...?**<br>ASK EACH AND THEN ADD TO CALCULATE THE TOTAL.	FOR INDIVIDUAL: **Your average monthly income from:**<br>**Employment**: _______________<br>**Money given by someone in your family, a friend, or your [spouse /partner /boyfriend /girlfriend]:**<br>___________<br>**Your grants** (INCLUDES GRANT FOR HER CHILD)**:** ___________<br>**Other sources**: __________________<br>Total: _______________________
b. **I'd also like to know the average monthly income for the other people that live in your house. Again, this helps us to understand whether getting health care is a burden for you and your household. Can you tell me...?**<br>ASK EACH AND THEN ADD TO CALCULATE THE TOTAL. INDICATE D/K IF NOT KNOWN FOR ANY.	FOR THE REST OF THE HOUSEHOLD: **The average monthly income for the other people in your house from:**<br>**Employment**: _______________<br>**Money given to them by a family member, friend, or a [spouse /partner /boyfriend/girlfriend]:**<br>___________<br>**Their grants:** ___________<br>**Other sources**: __________________<br>Total: _______________________

Do Not Circulate

Today's Date					
d	d	m	m	y	y

Study ID				

Part B: Health conditions, service quality

Now I'd like to ask you some questions about your health and health-related needs.	
**17. Do you have any health conditions or health-related problems?** TICK ALL THAT APPLY	❑ 0 = No/none<br>❑ 1 = Hypertension/high blood pressure<br>❑ 2 = Diabetes<br>❑ 3 = Cancer, type: __________<br>❑ 4 = Lung disease<br>❑ 5 = Mental health problem(s)<br>❑ 6 = VIH<br>❑ 7 = TB<br>❑ 8 = Other: _________<br>❑ 9 = Other: _________<br>❑ 10 = Other: _________<br>❑ 11 = Other: __________
SKIP IF VIH IS MENTIONED IN Q17<br><br>**18.** a. **Do you know your VIH status?**<br><br>b. IF KNOWN, **What is your VIH status?**	☐ 0 = No → GO TO Q19<br>☐ 1 =Yes<br>☐ 2 = Refused<br><br>☐ 1 = Negative<br>☐ 2 = Positive<br>☐ 3 = Refused
**19. a) How many times did you visit a clinic or hospital for any reason in the past year? This could include a visit for your child or children.** DO NOT INCLUDE TODAY'S VISIT<br><br>**b)** IF VISITED A CLINIC OR HOSPITAL, **What did you go for?** MARK ALL THAT APPLY. DO NOT INCLUDE TODAY'S VISIT.	❑ 0 times / didn't go → GO TO Q20<br>❑ 1<br>❑ 2<br>❑ 3<br>❑ 4<br>❑ 5<br>❑ 6<br>❑ 7<br>❑ 8<br>❑ 9<br>❑ 10<br>❑ >10<br><br>❑ 1 = Hypertension/high blood pressure<br>❑ 2 = Diabetes<br>❑ 3 = Cancer, type: __________<br>❑ 4 = Lung disease<br>❑ 5 = Mental health problem(s)<br>❑ 6 = VIH<br>❑ 7 = TB<br>❑ 8 = Contraception<br>❑ 9 = Other: _________<br>❑ 10 = Other: _________<br>❑ 11 = Other: _________<br>❑ 12 = Other: _________

Do Not Circulate

Today's Date					
d	d	m	m	y	y

Study ID				

---

21. I'd like to know what is important for you when you think about the quality of the health care you get. I'm going to list a few things that some people think are important. I'd like to know which are the <u>three most important things</u> for you when you think about health service quality. LIST ALL ITEMS AND THEN ASK WHICH ARE THE THREE MOST IMPORTANT. SHOW VISUAL AID

	Indicate top three most important items
a. Travel time/distance to the facility	❑
b. Waiting time at the facility	❑
c. Cost of services	❑
d. Friendly staff at the facility	❑
e. Knowledgeable staff	❑
f. Getting all the information you want or need	❑
g. Confidentiality	❑
h. Getting everything you need in one visit	❑
i. Cleanliness at the facility	❑

Part C: Sexual and Reproductive Health Needs Assessment and Costs

Do Not Circulate

Now I'd like to ask you some questions about your sexual and reproductive health. Sexual and reproductive health includes things like your relationships, pregnancy, having children, women's health issues and even violence against women.

I want to remind you that everything we discuss will be kept confidential. Some of the questions might be about private or personal information, but I'm not here to judge you. I want to know more about these things so that we can better understand the kind of health care that women like you might need.

I hope that the questions don't make you feel uncomfortable. You can decline to answer if you want to. Also, I'm not a doctor, nurse or social worker. If for some reason you want to talk to a doctor, nurse or social worker about anything after our interview is over, I can refer you to someone here or give you a number for another place that's convenient for you.

**22.** a. **Have you ever had sex?**	☐ 0 = No → GO TO Q25<br>☐ 1 =Yes
b. IF YES, **Have you had sex in the past 12 months?**	☐ 0 = No<br>☐ 1 = Yes

Today's Date					
d	d	m	m	y	y

Study ID				

FERTILITY / INFERTILITY NEEDS AND COSTS

**23. a. How many times have you been pregnant in your lifetime?**	NUMBER: _____ IF 0, →GO TO Q23C.
**b. When was the last time you were pregnant?** INDICATE WHEN THE PREGNANCY ENDED	❑ 1 = Currently pregnant<br>❑ 2 = In the last year (0-12 months ago)<br>❑ 3 = More than one year ago
**c. Have you tried to become pregnant during the last year?**	☐ 0 = No → GO TO Q25<br>☐ 1 =Yes
**d. Did you experience any problems while trying to fall pregnant?** IF YES, DESCRIBE.	☐ 0 = No → GO TO Q25<br>☐ 1 =Yes, Describe__________<br>__________________________
**e. Did you try to get help for your problem?**	☐ 0 = No<br>☐ 1 =Yes → GO TO Q23g
**f.** If e=NO, **Why not?** ASK, THEN GO TO Q25.	*OPEN RESPONSE:*
**g. IF e= YES, How many place(s) did you go to for help?**	☐ 1<br>☐ 2<br>☐ 3<br>☐ >3 COMPLETE Q24 FOR THE LAST THREE PLACES SHE WENT.

Do Not Circulate

Today's Date					
d	d	m	m	y	y

Study ID				

	PLACE 1	PLACE 2	PLACE 3
**24. a. When you were seeking help for your fertility problems, what kinds of place(s) did you go to?**	☐ 1 = Public clinic/hospital<br>☐ 2 = Private clinic/hospital<br>☐ 3 = Pharmacy/chemist<br>☐ 4 = Family/relative/friend<br>☐ 5 = Partner<br>☐ 6 =Traditional healer<br>☐ 7 = Other, ________	☐ 1 = Public clinic/hospital<br>☐ 2 = Private clinic/hospital<br>☐ 3 = Pharmacy/chemist<br>☐ 4 = Family/relative/friend<br>☐ 5 = Partner<br>☐ 6 =Traditional healer<br>☐ 7 = Other, ________	☐ 1 = Public clinic/hospital<br>☐ 2 = Private clinic/hospital<br>☐ 3 = Pharmacy/chemist<br>☐ 4 = Family/relative/friend<br>☐ 5 = Partner<br>☐ 6 =Traditional healer<br>☐ 7 = Other, ________
**b. Where was that place?**	NAME: ________________<br>CODE: ________________	NAME: ________________<br>CODE: ________________	NAME: ________________<br>CODE: ________________
**i. Was it close to home?**	☐ 0 = No ☐ 1 = Yes	☐ 0 = No ☐ 1 = Yes	☐ 0 = No ☐ 1 = Yes
**ii. Was it at or close to work?**	☐ 0 = No<br>☐ 1 = Yes<br>☐ 2 = N/A, not working	☐ 0 = No<br>☐ 1 = Yes<br>☐ 2 = N/A, not working	☐ 0 = No<br>☐ 1 = Yes<br>☐ 2 = N/A, not working
**c. How many times did you go to that place?**	______	______	______

Do Not Circulate

**Today's Date**					
d	d	m	m	y	y

**Study ID**				

	**PLACE 1**	**PLACE 2**	**PLACE 3**
**d. Where did you eventually get the help?** MARK ALL THAT APPLY.	❑ 1 = At place 1<br>❑ 4 = I didn't get help	❑ 2 = At place 2	❑ 3 = At place 3
**e. If you traveled from home, how would you travel to that place?**	❑ 1 = Walked<br>❑ 2 = Public transport<br>❑ 3 = Private taxi<br>❑ 4 = Friend/family lift<br>❑ 5 = Drove own car<br>❑ 6 = Other	❑ 1 = Walked<br>❑ 2 = Public transport<br>❑ 3 = Private taxi<br>❑ 4 = Friend/family lift<br>❑ 5 = Drove own car<br>❑ 6 = Other	❑ 1 = Walked<br>❑ 2 = Public transport<br>❑ 3 = Private taxi<br>❑ 4 = Friend/family lift<br>❑ 5 = Drove own car<br>❑ 6 = Other
**f. On average, how long would it take to travel to that place (one way) from the place where you stay?**	______ : ______<br>h h m m	______ : ______<br>h h m m	______ : ______<br>h h m m
**g. How much is the transport from the place where you stay to that place (one way)?**	ZAR ______ . _____	ZAR ______ . _____	ZAR ______ . _____
**h. How long did you spend at that place including waiting time, seeing the nurse/doctor, etc?**	______ : ______<br>h h m m	______ : ______<br>h h m m	______ : ______<br>h h m m
i. **How would you rate the quality of care that you had at the place where you got help for your fertility problems?** SHOW VISUAL AID	❑ 1 = Very good<br>❑ 2 = Good<br>❑ 3 = Neutral<br>❑ 4 = Fair<br>❑ 5 = Very bad	❑ 1 = Very good<br>❑ 2 = Good<br>❑ 3 = Neutral<br>❑ 4 = Fair<br>❑ 5 = Very bad	❑ 1 = Very good<br>❑ 2 = Good<br>❑ 3 = Neutral<br>❑ 4 = Fair<br>❑ 5 = Very bad

Do Not Circulate

Today's Date					
d	d	m	m	y	y

Study ID				

**j. Why is that?**	OPEN RESPONSE:	OPEN RESPONSE:	OPEN RESPONSE:

**k. Did you miss work because of your efforts to get help for your fertility problems? [**IF MORE THAN ONE PLACE: **This could be because of visits to any of the places you mentioned.]**<br><br>**l.** IF YES**, How many days?**	☐ 0= No → GO TO M<br>❑ 1 = Yes<br><br>______ days
**m. Did you lose any income from work or other sources because you had to get help for your fertility problems? [**IF MORE THAN ONE PLACE: **This could be because of visits to any of the places you mentioned.]**<br><br>**n.** IF YES, **How much?**	❑ 0= No → GO TO O<br>❑ 1 = Yes<br><br>ZAR _________ . ________
**o. Were you unable to complete some of your daily tasks because you were getting help for your fertility problems? [**IF MORE THAN ONE PLACE: **This could be because of visits to any of the places you mentioned.]**	❑ 0= No<br>❑ 1 = Yes

Do Not Circulate

Today's Date					
d	d	m	m	y	y

Study ID				

Do Not Circulate

**I'd like to know about your expenses related to getting help for your fertility problems. [Considering all the places where you went,] did you have to pay for...**	
p. **...<u>medications</u>?** IF YES, **How much?**	☐ 0= No<br>❑ 1 = Yes ZAR _____ . ______
**q. ... <u>consultation</u>(s) or doctor's fee(s)?** IF YES, **How much?**	☐ 0= No<br>❑ 1 = Yes ZAR _____ . ______
**r. ...<u>child care</u> while you were getting help for your fertility problems?** IF YES, **How much?**	☐ 0= No<br>❑ 1 = Yes ZAR _____ . ______<br>❑ 2 = N/A (no children)
**s. ...<u>other expenses</u> related to getting help for your fertility problems?**	☐ 0= No → GO TO T<br>❑ 1 = Yes
i. IF YES, **What kinds of things did you pay for?**	LIST: ____________________
ii. AND IF YES, **How much did you pay for all of those expenses in total?**	ZAR _________ . ________
**t. Did anyone help you to pay for your expenses (e.g. transport, fees, etc)?**	☐ 0= No → GO TO Q25<br>❑ 1 = Yes<br>❑ 2 = N/A (no expenses)
u. IF YES, **how did they help?**	OPEN RESPONSE:

Today's Date					
d	d	m	m	y	y

Study ID				

CONTRACEPTIVE NEEDS

**25.** a. **Do you have children?** [COUNT BIOLOGICAL CHILDREN ONLY.] b. IF YES, **How many?**	☐ 0 = No<br>☐ 1 =Yes, NUMBER: _____
**26. a. Would you like to have a(nother) child or would you prefer to not have any (more) children?**	❑ 1 = Wants to have a(nother) child<br>❑ 2 = Doesn't want more/any children →GO TO Q27<br>❑ 3 = Not applicable - Can't get pregnant →GO TO Q27<br>❑ 4 = Undecided/doesn't know →GO TO Q27
b. **Would you like to fall pregnant (again) any time in the next two years/24 months?**	❑ 0 = No<br>❑ 1 = Yes<br>❑ 3 = Undecided/doesn't know

27. I'd like to ask you some questions about contraception, or family planning. I'm going to read through a list of modern methods and more traditional ways of preventing or delaying pregnancy.

Do Not Circulate

- **a. Can you tell me if you've ever heard of...**READ OPTION ALOUD.
- **b.** IF a=YES, **And have you (or your partner or partners) <u>ever</u> used that method to delay or avoid getting pregnant?**
- **c.** IF b=YES, **And are you (or your partner) <u>currently</u> using that method to delay or avoid getting pregnant?**

TICK ALL THAT APPLY FOR A-C. N= No, Y = Yes.

	**a. Ever heard of...**	**b. Ever used...**	**c. Currently using**
Modern methods:<br>1= **Female sterilization**	❑ 0 = N ❑ 1 = Y	❑ 0 = N ❑ 1 =Y<br>❑ 2 = N/A	❑ 0 = N ❑ 1=Y<br>❑ 2 = N/A
2= **Male sterilization**	❑ 0 = N ❑ 1 = Y	❑ 0 = N ❑ 1 =Y<br>❑ 2 = N/A	❑ 0 = N ❑ 1=Y<br>❑ 2 = N/A
3= **IUD – Copper**	❑ 0 = N ❑ 1 = Y	❑ 0 = N ❑ 1 =Y<br>❑ 2 = N/A	❑ 0 = N ❑ 1=Y<br>❑ 2 = N/A
4 = **IUD – Mirena**	❑ 0 = N ❑ 1 = Y	❑ 0 = N ❑ 1 =Y<br>❑ 2 = N/A	❑ 0 = N ❑ 1=Y<br>❑ 2 = N/A
5 = **Injectable – Depot** (3 month)	❑ 0 = N ❑ 1 = Y	❑ 0 = N ❑ 1 =Y<br>❑ 2 = N/A	❑ 0 = N ❑ 1=Y<br>❑ 2 = N/A

Today's Date					
d	d	m	m	y	y

Study ID				

	a. Ever heard of...	b. Ever used...	c. Currently using
6 = **Injectable – Nuristerate** (2 month)	❑ 0 = N ❑ 1 = Y	❑ 0 = N ❑ 1 =Y ❑ 2 = N/A	❑ 0 = N ❑ 1=Y ❑ 2 = N/A
7 = **Subdermal implant – Norplant, Jadelle**	❑ 0 = N ❑ 1 = Y	❑ 0 = N ❑ 1 =Y ❑ 2 = N/A	❑ 0 = N ❑ 1=Y ❑ 2 = N/A
8 = **Pills**	❑ 0 = N ❑ 1 = Y	❑ 0 = N ❑ 1 =Y ❑ 2 = N/A	❑ 0 = N ❑ 1=Y ❑ 2 = N/A
9 = **Contraceptive ring**	❑ 0 = N ❑ 1 = Y	❑ 0 = N ❑ 1 =Y ❑ 2 = N/A	❑ 0 = N ❑ 1=Y ❑ 2 = N/A
10 = **Contraceptive patch**	❑ 0 = N ❑ 1 = Y	❑ 0 = N ❑ 1 =Y ❑ 2 = N/A	❑ 0 = N ❑ 1=Y ❑ 2 = N/A
11 = **Male condoms**	❑ 0 = N ❑ 1 = Y	❑ 0 = N ❑ 1 =Y ❑ 2 = N/A	❑ 0 = N ❑ 1=Y ❑ 2 = N/A
12 = **Female condoms**	❑ 0 = N ❑ 1 = Y	❑ 0 = N ❑ 1 =Y ❑ 2 = N/A	❑ 0 = N ❑ 1=Y ❑ 2 = N/A
13 = **Emergency contraception**	❑ 0 = N ❑ 1 = Y	❑ 0 = N ❑ 1 =Y ❑ 2 = N/A	❑ 0 = N ❑ 1=Y ❑ 2 = N/A
Traditional methods:<br>14 = **Standard days / Rhythm method**	❑ 0 = N ❑ 1 = Y	❑ 0 = N ❑ 1 =Y ❑ 2 = N/A	❑ 0 = N ❑ 1=Y ❑ 2 = N/A
15 = **Lactational ammenorhea**	❑ 0 = N ❑ 1 = Y	❑ 0 = N ❑ 1 =Y ❑ 2 = N/A	❑ 0 = N ❑ 1=Y ❑ 2 = N/A
16 = **Withdrawal**	❑ 0 = N ❑ 1 = Y	❑ 0 = N ❑ 1 =Y ❑ 2 = N/A	❑ 0 = N ❑ 1=Y ❑ 2 = N/A
Other<br>17 = **Any other method** SPECIFY: ______________	❑ 0 = N ❑ 1 = Y	❑ 0 = N ❑ 1 =Y ❑ 2 = N/A	❑ 0 = N ❑ 1=Y ❑ 2 = N/A

Do Not Circulate

Today's Date					
d	d	m	m	y	y

Study ID				

Q28 IS FOR WOMEN WHO ARE NOT USING A METHOD. SKIP Q28 IF SHE OR HER PARTNER IS CURRENTLY USING ANY METHOD.	
**28. a. You said you're not currently using any contraceptive method(s). Would you like to try using a contraceptive method in the future?**	❑ 0= No<br>❑ 1 = Yes<br>❑ 2 = Don't know
b. **Why is that?** (PROBE: IF a=YES, which method?)	OPEN RESPONSE:
c. **Do you feel that you have enough information about all the methods so that you can request the one that is right for you?**	❑ 0= No<br>❑ 1 = Yes<br>❑ 2 = Don't know
Q29 IS FOR WOMEN WHO ARE CURRENTLY USING A METHOD. SKIP Q29 IF SHE OR HER PARTNER IS NOT CURRENTLY USING ANY METHOD.	
**29. a. Are you happy with your current contraceptive method(s)?**	❑ 0= No<br>❑ 1 = Yes<br>❑ 2 = Don't know
**b. Why is that?** PROBE WHY SHE IS OR IS NOT HAPPY WITH THE METHOD(S).	OPEN RESPONSE:
**c. Would you like to try using a different method in the future?**	☐ 0= No<br>❑ 1 = Yes<br>❑ 2 = Don't know
d. **Why is that?** (PROBE: IF c=YES, which method?)	OPEN RESPONSE:
e. **Do you feel that you have enough information about all the methods so that you can request the one that is right for you?**	❑ 0= No<br>❑ 1 = Yes<br>❑ 2 = Don't know

Do Not Circulate

Today's Date					
d	d	m	m	y	y

Study ID				

---

30. I'd like to know what is important for you when you think about the quality of contraceptive services. I'm going to list a few things that some people think are important. I'd like to know which are the <u>three most important things</u> for you when you think about health contraceptive service quality. LIST ALL ITEMS AND THEN ASK WHICH ARE THE THREE MOST IMPORTANT. SHOW VISUAL AID

	Indicate top three most important items
a. Travel time/distance to the facility	❑
b. Waiting time at the facility	❑
c. Cost of services	❑
d. Friendly staff at the facility	❑
e. Knowledgeable staff	❑
f. Getting all the information you want or need	❑
g. Confidentiality	❑
h. Getting everything you need in one visit	❑
i. Cleanliness at the facility	❑
j. Having a choice of methods	❑

CONTRACEPTIVE COSTS

SKIP Q31-32 IF SHE OR HER PARTNER IS <u>NOT</u> CURRENTLY USING ANY MODERN METHOD. ALSO, IF HER MAIN METHOD IS OBTAINED BY HER PARTNER (E.G. MALE CONDOMS OR VASECTOMY), SKIP Q31-32.

Do Not Circulate

**31.** a. 1 **When was the last time you got your [main] contraceptive method?** IF SHE USES MORE THAN ONE NOW, ASK ABOUT HER MOST EFFECTIVE METHOD AND LIST THAT METHOD HERE:<br><br>---	☐ 1 = In the last year<br>☐ 2 = More than one year ago → GO TO Q33<br>☐ 3 = Can't remember → GO TO Q33
a 2 **How many times did you refill your method in the last year?**	NUMBER: ____________
b. **The last time you got your [main] current contraceptive method, how many place(s) did you go to?**	☐ 1<br>☐ 2<br>☐ 3<br>☐ >3 COMPLETE Q32 FOR THE LAST THREE PLACES SHE WENT.

**Today's Date**					
d	d	m	m	y	y

**Study ID**				

Do Not Circulate

	**PLACE 1**	**PLACE 2**	**PLACE 3**
**32. a. The last time you got your [main] current contraceptive method, what kinds of place(s) did you go to?**	☐ 1 = Public clinic/hospital<br>☐ 2 = Private clinic/hospital<br>☐ 3 = Pharmacy/chemist<br>☐ 4 = Family/relative/friend<br>☐ 5 = Partner<br>☐ 6 =Traditional healer<br>☐ 7 = Other, _______	☐ 1 = Public clinic/hospital<br>☐ 2 = Private clinic/hospital<br>☐ 3 = Pharmacy/chemist<br>☐ 4 = Family/relative/friend<br>☐ 5 = Partner<br>☐ 6 =Traditional healer<br>☐ 7 = Other, _______	☐ 1 = Public clinic/hospital<br>☐ 2 = Private clinic/hospital<br>☐ 3 = Pharmacy/chemist<br>☐ 4 = Family/relative/friend<br>☐ 5 = Partner<br>☐ 6 =Traditional healer<br>☐ 7 = Other, _______
**b. Where was that place?**	NAME: ____________<br>CODE: ____________	NAME: ____________<br>CODE: ____________	NAME: ____________<br>CODE: ____________
**i. Was it close to home?**	☐ 0 = No ☐ 1 = Yes	☐ 0 = No ☐ 1 = Yes	☐ 0 = No ☐ 1 = Yes
**ii. Was it at or close to work?**	☐ 0 = No<br>☐ 1 = Yes<br>☐ 2 = N/A, not working	☐ 0 = No<br>☐ 1 = Yes<br>☐ 2 = N/A, not working	☐ 0 = No<br>☐ 1 = Yes<br>☐ 2 = N/A, not working
**c. How many times did you go to that place?**	______	______	______
**d. Where did you eventually get the method?** MARK ALL THAT APPLY	☐ 1 = At place 1<br>☐ 4 = I didn't get help	☐ 2 = At place 2	☐ 3 = At place 3
	**PLACE 1**	**PLACE 2**	**PLACE 3**

Today's Date					
d	d	m	m	y	y

Study ID				

**e. If you traveled from home, how would you travel to that place?** MARK ALL THAT APPLY	❑ 1 = Walked<br>❑ 2 = Public transport<br>❑ 3 = Private taxi<br>❑ 4 = Friend/family lift<br>❑ 5 = Drove own car<br>❑ 6 = Other	❑ 1 = Walked<br>❑ 2 = Public transport<br>❑ 3 = Private taxi<br>❑ 4 = Friend/family lift<br>❑ 5 = Drove own car<br>❑ 6 = Other	❑ 1 = Walked<br>❑ 2 = Public transport<br>❑ 3 = Private taxi<br>❑ 4 = Friend/family lift<br>❑ 5 = Drove own car<br>❑ 6 = Other
**f. On average, how long would it take to travel to that place (one way) from the place where you stay?**	______ : ______<br>h h m m	______ : ______<br>h h m m	______ : ______<br>h h m m
**g. How much is the transport from the place where you stay to that place (one way)?**	ZAR ______ . _____	ZAR ______ . _____	ZAR ______ . _____
**h. How long did you spend at that place including waiting time, [getting the method], etc?**	______ : ______<br>h h m m	______ : ______<br>h h m m	______ : ______<br>h h m m
**i. How would you rate the quality of care that you had at the place(s) where you went for your method?** SHOW VISUAL AID<br><br>**j. Why is that?**	☐ 1 = Very good<br>☐ 2 = Good<br>☐ 3 = Neutral<br>☐ 4 = Fair<br>☐ 5 = Very bad<br><br>OPEN RESPONSE:	☐ 1 = Very good<br>☐ 2 = Good<br>☐ 3 = Neutral<br>☐ 4 = Fair<br>☐ 5 = Very bad<br><br>OPEN RESPONSE:	☐ 1 = Very good<br>☐ 2 = Good<br>☐ 3 = Neutral<br>☐ 4 = Fair<br>☐ 5 = Very bad<br><br>OPEN RESPONSE:

**k. Did you miss work while you were getting your method? [**IF MORE THAN ONE PLACE: **This could be because of visits to any of the places you mentioned.]**	☐ 0= No → GO TO M<br>❑ 1 = Yes

Do Not Circulate

Today's Date					
d	d	m	m	y	y

Study ID				

**l.** IF YES**, How many days?**	______ days
**m. Did you lose any income from work or other sources because you had to get your method? [**IF MORE THAN ONE PLACE: **This could be because of visits to any of the places you mentioned.]**<br><br>**n.** IF YES, **How much?**	☐ 0= No → GO TO O<br>☐ 1 = Yes<br><br>ZAR _________ . ________
**o. Were you unable to complete some of your daily tasks because you were getting your method? [**IF MORE THAN ONE PLACE: **This could be because of visits to any of the places you mentioned.]**	❑ 0= No<br>❑ 1 = Yes
**I'd like to know about your expenses related to getting your method. [Considering all the places where you went,] did you have to pay for:**<br><br>p. **...the method?** IF YES, **How much?**<br><br>**q. ... consultation(s) or doctor's fee(s)?** IF YES**, How much?**<br><br>**r. ...child care while you were getting the method?** IF YES, **How much?**<br><br>**s. ...other expenses related to getting the method?**	☐ 0= No<br>❑ 1 = Yes ZAR _____ . ______<br><br>☐ 0= No<br>❑ 1 = Yes ZAR _____ . ______<br><br>☐ 0= No<br>❑ 1 = Yes ZAR _____ . ______<br>❑ 2 = N/A (no children)<br><br>☐ 0= No → GO TO T<br>❑ 1 = Yes

Do Not Circulate

Today's Date					
d	d	m	m	y	y

Study ID				

i. IF YES, **What kinds of things did you pay for?**<br><br>ii. AND IF YES, **How much did you pay for all of those expenses in total?**	LIST: ____________________<br><br>ZAR _________ . ________
**t. Did anyone help you to pay for your expenses (e.g. transport, fees, etc)?**<br><br>u. IF YES, **how did they help?**	☐ 0= No → GO TO Q33<br>☐ 1 = Yes<br>☐ 2 = N/A (no expenses)<br><br>OPEN RESPONSE:

Do Not Circulate

MENSTRUATION NEEDS AND COSTS

**33. a. Have you experienced any menstrual problems in the past year?**<br><br>**b.** IF a=YES, **Did you seek help for those problems?**	☐ 0= No → GO TO Q35<br>☐ 1 = Yes, DESCRIBE:<br>____________________________<br><br>☐ 0= No<br>☐ 1 = Yes → GO TO Q33d<br><br>OPEN RESPONSE

Today's Date					
d	d	m	m	y	y

Study ID				

**c.** If b=NO, **Why not?** PROVIDE RESPONSE AND THEN GO TO Q35.	
**d.** IF b= YES, **How many place(s) did you go to for help?**	☐ 1<br>☐ 2<br>☐ 3<br>☐ >3 COMPLETE Q34 FOR THE LAST THREE PLACES SHE WENT.

Do Not Circulate

	**PLACE 1**	**PLACE 2**	**PLACE 3**
**34. a. When you were seeking help for your menstrual problems, what kinds of place(s) did you go to?**	☐ 1 = Public clinic/hospital<br>☐ 2 = Private clinic/hospital<br>☐ 3 = Pharmacy/chemist<br>☐ 4 = Family/relative/friend<br>☐ 5 = Partner<br>☐ 6 =Traditional healer<br>☐ 7 = Other, _______	☐ 1 = Public clinic/hospital<br>☐ 2 = Private clinic/hospital<br>☐ 3 = Pharmacy/chemist<br>☐ 4 = Family/relative/friend<br>☐ 5 = Partner<br>☐ 6 =Traditional healer<br>☐ 7 = Other, _______	☐ 1 = Public clinic/hospital<br>☐ 2 = Private clinic/hospital<br>☐ 3 = Pharmacy/chemist<br>☐ 4 = Family/relative/friend<br>☐ 5 = Partner<br>☐ 6 =Traditional healer<br>☐ 7 = Other, _______
**b. Where was that place?**	NAME: _______________<br>CODE: _______________	NAME: _______________<br>CODE: _______________	NAME: _______________<br>CODE: _______________
**i. Was it close to home?**	☐ 0 = No ☐ 1 = Yes	☐ 0 = No ☐ 1 = Yes	☐ 0 = No ☐ 1 = Yes
**ii. Was it at or close to work?**	☐ 0 = No<br>☐ 1 = Yes<br>☐ 2 = N/A, not working	☐ 0 = No<br>☐ 1 = Yes<br>☐ 2 = N/A, not working	☐ 0 = No<br>☐ 1 = Yes<br>☐ 2 = N/A, not working

Today's Date					
d	d	m	m	y	y

Study ID				

**c. How many times did you go to that place?**	_______	_______	_______
**d. Where did you eventually get the help?** MARK ALL THAT APPLY	☐ 1 = At place 1<br>☐ 4 = I didn't get help	☐ 2 = At place 2	☐ 3 = At place 3
**e. If you traveled from home, how would you travel to that place?** MARK ALL THAT APPLY	☐ 1 = Walked<br>☐ 2 = Public transport<br>☐ 3 = Private taxi<br>☐ 4 = Friend/family lift<br>☐ 5 = Drove own car<br>☐ 6 = Other	☐ 1 = Walked<br>☐ 2 = Public transport<br>☐ 3 = Private taxi<br>☐ 4 = Friend/family lift<br>☐ 5 = Drove own car<br>☐ 6 = Other	☐ 1 = Walked<br>☐ 2 = Public transport<br>☐ 3 = Private taxi<br>☐ 4 = Friend/family lift<br>☐ 5 = Drove own car<br>☐ 6 = Other
**f. On average, how long would it take to travel to that place (one way) from the place where you stay?**	_______ : _______<br>h h m m	_______ : _______<br>h h m m	_______ : _______<br>h h m m
**g. How much is the transport from the place where you stay to that place (one way)?**	ZAR ______ . _____	ZAR ______ . _____	ZAR ______ . _____
**h. How long did you spend at that place including waiting time, seeing the nurse/doctor, etc?**	_______ : _______<br>h h m m	_______ : _______<br>h h m m	_______ : _______<br>h h m m
i. **How would you rate the quality of care that you had at the place(s) where you went for your menstrual problems?** SHOW VISUAL AID	☐ 1 = Very good<br>☐ 2 = Good<br>☐ 3 = Neutral<br>☐ 4 = Fair<br>☐ 5 = Very bad	☐ 1 = Very good<br>☐ 2 = Good<br>☐ 3 = Neutral<br>☐ 4 = Fair<br>☐ 5 = Very bad	☐ 1 = Very good<br>☐ 2 = Good<br>☐ 3 = Neutral<br>☐ 4 = Fair<br>☐ 5 = Very bad

Do Not Circulate

Today's Date					
d	d	m	m	y	y

Study ID				

**j. Why is that?**	OPEN RESPONSE:	OPEN RESPONSE:	OPEN RESPONSE:

**k. Did you miss work because of your efforts to get help for your menstrual problems? [**IF MORE THAN ONE PLACE: **This could be because of visits to any of the places you mentioned.]**<br><br>**l.** IF YES**, How many days?**	☐ 0= No → GO TO M<br>❑ 1 = Yes<br><br>______ days
**m. Did you lose any income from work or other sources because you had to get help for your menstrual problems? [**IF MORE THAN ONE PLACE: **This could be because of visits to any of the places you mentioned.]**<br><br>**n.** IF YES, **How much?**	☐ 0= No → GO TO O<br>❑ 1 = Yes<br><br>ZAR _________ . ________
**o. Were you unable to complete some of your daily tasks because you were getting help for your menstrual problems? [**IF MORE THAN ONE PLACE: **This could be because of visits to any of the places you mentioned.]**	❑ 0= No<br>❑ 1 = Yes
**I'd like to know about your expenses related to getting help for your menstrual problems. [Considering all the places where you went,] did you have to pay for...**<br><br>p. **...<u>medications</u>?** IF YES, **How much?**<br><br>**q. ... <u>consultation</u>(s) or doctor's fee(s)?** IF YES**, How much?**	 <br>☐ 0= No<br>❑ 1 = Yes ZAR _____ . ______<br><br>☐ 0= No<br>❑ 1 = Yes ZAR _____ . ______

Do Not Circulate

Today's Date					
d	d	m	m	y	y

Study ID				

**r. ...<u>child care</u> while you were getting help for your menstrual problems?** IF YES, **How much?**	❑ 0= No<br>❑ 1 = Yes ZAR _____ . ______<br>❑ 2 = N/A (no children)
**s. ...<u>other expenses</u> related to getting help for your menstrual problems?**	❑ 0= No → GO TO T<br>❑ 1 = Yes
i. IF YES, **What kinds of things did you pay for?**	LIST: _________________
ii. AND IF YES, **How much did you pay for all of those expenses in total?**	ZAR ________ . ________
**t. Did anyone help you to pay for your expenses (e.g. transport, fees, etc)?**	❑ 0= No → GO TO Q35<br>❑ 1 = Yes<br>❑ 2 = N/A (no expenses)
u. IF YES, **how did they help?**	OPEN RESPONSE:

Do Not Circulate

MENOPAUSE NEEDS AND COSTS

**35. a. 1 Have you experienced any menopausal symptoms in the past year?**	❑ 0 = No → GO TO Q37<br>❑ 1 = Yes<br>❑ 2 = Don't know, not sure → GO TO Q37<br><br>IF YES, TICK ALL THAT APPLY:

Today's Date					
d	d	m	m	y	y

Study ID				

Do Not Circulate

a 2. IF YES, **What symptoms did you have?** TICK ALL THAT APPLY.	☐ 1 = Hot flashes<br>☐ 2 = Night sweats<br>☐ 3 = Irregular period<br>☐ 4 = Vaginal dryness<br>☐ 5 = Mood swings<br>☐ 6 = Loss of libido<br>☐ 7 = Memory Loss<br>☐ 8 = Other, specify: ____________________
**b.** IF a1=YES, **Did you seek help for those symptoms?**	☐ 0= No<br>☐ 1 = Yes → GO TO Q35d
c. If b=NO, **Why not?** PROVIDE RESPONSE AND THEN GO TO Q37	OPEN RESPONSE:
**d.** IF b= YES, **How many place(s) did you go to for help?**	☐ 1<br>☐ 2<br>☐ 3<br>☐ >3 COMPLETE Q36 FOR THE LAST THREE PLACES SHE WENT TO.

Today's Date					
d	d	m	m	y	y

Study ID				

	Place 1	Place 2	Place 3
**36. a. When you were seeking help for your menopause symptoms, what kinds of place(s) did you go to?**	☐ 1 = Public clinic/hospital<br>☐ 2 = Private clinic/hospital<br>☐ 3 = Pharmacy/chemist<br>☐ 4 = Family/relative/friend<br>☐ 5 = Partner<br>☐ 6 =Traditional healer<br>☐ 7 = Other, _______	☐ 1 = Public clinic/hospital<br>☐ 2 = Private clinic/hospital<br>☐ 3 = Pharmacy/chemist<br>☐ 4 = Family/relative/friend<br>☐ 5 = Partner<br>☐ 6 =Traditional healer<br>☐ 7 = Other, _______	☐ 1 = Public clinic/hospital<br>☐ 2 = Private clinic/hospital<br>☐ 3 = Pharmacy/chemist<br>☐ 4 = Family/relative/friend<br>☐ 5 = Partner<br>☐ 6 =Traditional healer<br>☐ 7 = Other, _______
**b. Where was that place?**	NAME: ________________<br>CODE: ________________	NAME: ________________<br>CODE: ________________	NAME: ________________<br>CODE: ________________
**i. Was it close to home?**	☐ 0 = No ☐ 1 = Yes	☐ 0 = No ☐ 1 = Yes	☐ 0 = No ☐ 1 = Yes
**ii. Was it at or close to work?**	☐ 0 = No<br>☐ 1 = Yes<br>☐ 2 = N/A, not working	☐ 0 = No<br>☐ 1 = Yes<br>☐ 2 = N/A, not working	☐ 0 = No<br>☐ 1 = Yes<br>☐ 2 = N/A, not working
**c. How many times did you go to that place?**	______	______	______
**d. Where did you eventually get the help?** MARK ALL THAT APPLY	☐ 1 = At place 1<br>☐ 4 = I didn't get help	☐ 2 = At place 2	☐ 3 = At place 3

Do Not Circulate

Today's Date					
d	d	m	m	y	y

Study ID				

Do Not Circulate

	Place 1	Place 2	Place 3
**e. If you traveled from home, how would you travel to that place?** MARK ALL THAT APPLY	❑ 1 = Walked<br>❑ 2 = Public transport<br>❑ 3 = Private taxi<br>❑ 4 = Friend/family lift<br>❑ 5 = Drove own car<br>❑ 6 = Other	❑ 1 = Walked<br>❑ 2 = Public transport<br>❑ 3 = Private taxi<br>❑ 4 = Friend/family lift<br>❑ 5 = Drove own car<br>❑ 6 = Other	❑ 1 = Walked<br>❑ 2 = Public transport<br>❑ 3 = Private taxi<br>❑ 4 = Friend/family lift<br>❑ 5 = Drove own car<br>❑ 6 = Other
**f. On average, how long would it take to travel to that place (one way) from the place where you stay?**	______ : ______<br>h h m m	______ : ______<br>h h m m	______ : ______<br>h h m m
**g. How much is the transport from the place where you stay to that place (one way)?**	ZAR ______ . ______	ZAR ______ . ______	ZAR ______ . ______
**h. How long did you spend at that place including waiting time, seeing the doctor/nurse, etc?**	______ : ______<br>h h m m	______ : ______<br>h h m m	______ : ______<br>h h m m
**i. How would you rate the quality of care that you had at the place(s) where you went for your menopause symptoms?** SHOW VISUAL AID<br><br>**j. Why is that?**	☐ 1 = Very good<br>☐ 2 = Good<br>☐ 3 = Neutral<br>☐ 4 = Fair<br>☐ 5 = Very bad<br><br>OPEN RESPONSE:	☐ 1 = Very good<br>☐ 2 = Good<br>☐ 3 = Neutral<br>☐ 4 = Fair<br>☐ 5 = Very bad<br><br>OPEN RESPONSE:	☐ 1 = Very good<br>☐ 2 = Good<br>☐ 3 = Neutral<br>☐ 4 = Fair<br>☐ 5 = Very bad<br><br>OPEN RESPONSE:

Today's Date					
d	d	m	m	y	y

Study ID				

**k. Did you miss work because of your efforts to get help for your menopause symptoms? [**IF MORE THAN ONE PLACE: **This could be because of visits to any of the places you mentioned.]**<br><br>**l.** IF YES**, How many days?**	☐ 0= No → GO TO M<br>❑ 1 = Yes<br><br>______ days
**m. Did you lose any income from work or other sources because you had to get help for your menopause symptoms? [**IF MORE THAN ONE PLACE: **This could be because of visits to any of the places you mentioned.]**<br><br>**n.** IF YES, **How much?**	☐ 0= No → GO TO O<br>☐ 1 = Yes<br><br>ZAR _________ . ________
**o. Were you unable to complete some of your daily tasks because you were getting help for your menopause symptoms? [**IF MORE THAN ONE PLACE: **This could be because of visits to any of the places you mentioned.]**	☐ 0= No<br>❑ 1 = Yes
**I'd like to know about your expenses related to getting help for your menopause symptoms. [Considering all the places where you went,] did you have to pay for...**<br><br>p. **... <u>medications</u>?** IF YES, **How much?**<br><br>**q. ... <u>consultation</u>(s) or doctor's fee(s)?** IF YES**, How much?**<br><br>**r. ...<u>child care</u> while you were getting help for your menopause symptoms?** IF YES, **How much?**	 <br>☐ 0= No<br>❑ 1 = Yes ZAR _____ . ______<br><br>☐ 0= No<br>❑ 1 = Yes ZAR _____ . ______<br><br>☐ 0= No<br>❑ 1 = Yes ZAR _____ . ______<br>❑ 2 = N/A (no children)

Do Not Circulate

Today's Date					
d	d	m	m	y	y

Study ID				

**s. ...<u>other expenses</u> related to getting help for your menopause symptoms?**<br><br>i. IF YES, **What kinds of things did you pay for?**<br><br>ii. AND IF YES, **How much did you pay for all of those expenses in total?**	☐ 0= No → GO TO T<br>❑ 1 = Yes<br><br>LIST: ____________________<br><br>ZAR _________ . ________
**t. Did anyone help you to pay for your expenses (e.g. transport, fees, etc)?**<br><br>u. IF YES, **how did they help?**	☐ 0= No → GO TO Q37<br>❑ 1 = Yes<br>❑ 2 = N/A (no expenses)<br><br>OPEN RESPONSE:

Do Not Circulate

CERVICAL CANCER NEEDS AND COSTS

**37. a. Have you ever been screened for cervical cancer? Often that procedure is called a Pap smear.**<br><br>b. If a=NO, **Why not?** PROVIDE RESPONSE AND THEN GO TO Q39.<br><br>c. IF a=YES, **When was the last time you had a Pap?** IF SHE HAS HAD MORE THAN ONE, ASK ABOUT THE LAST TIME.	❑ 0 = No<br>❑ 1 = Yes → GO TO Q37c<br><br>OPEN RESPONSE:<br><br>☐ 1 = In the last year<br>☐ 2 = More than one year ago → GO TO Q39<br>☐ 3 = Can't remember → GO TO Q39

Today's Date					
d	d	m	m	y	y

Study ID				

**d. How many place(s) did you go to while trying to get that Pap?**	☐ 1<br>☐ 2<br>☐ 3<br>☐ >3 COMPLETE Q38 FOR THE LAST THREE PLACES SHE WENT.

	**Place 1**	**Place 2**	**Place 3**
**38. a. When you were trying to get your Pap smear, what kinds of place(s) did you go to?**	☐ 1 = Public clinic/hospital<br>☐ 2 = Private clinic/hospital<br>☐ 3 = Pharmacy/chemist<br>☐ 4 = Family/relative/friend<br>☐ 5 = Partner<br>☐ 6 =Traditional healer<br>☐ 7 = Other, _______	☐ 1 = Public clinic/hospital<br>☐ 2 = Private clinic/hospital<br>☐ 3 = Pharmacy/chemist<br>☐ 4 = Family/relative/friend<br>☐ 5 = Partner<br>☐ 6 =Traditional healer<br>☐ 7 = Other, _______	☐ 1 = Public clinic/hospital<br>☐ 2 = Private clinic/hospital<br>☐ 3 = Pharmacy/chemist<br>☐ 4 = Family/relative/friend<br>☐ 5 = Partner<br>☐ 6 =Traditional healer<br>☐ 7 = Other, _______
**b. Where was that place?**	NAME: ________________<br>CODE: ________________	NAME: ________________<br>CODE: ________________	NAME: ________________<br>CODE: ________________
**i. Was it close to home?**	☐ 0 = No ☐ 1 = Yes	☐ 0 = No ☐ 1 = Yes	☐ 0 = No ☐ 1 = Yes
**ii. Was it at or close to work?**	☐ 0 = No<br>☐ 1 = Yes<br>☐ 2 = N/A, not working	☐ 0 = No<br>☐ 1 = Yes<br>☐ 2 = N/A, not working	☐ 0 = No<br>☐ 1 = Yes<br>☐ 2 = N/A, not working
**c. How many times did you go to that place?**	______	______	______
d. **Where did you eventually get the help?** MARK ALL THAT APPLY	☐ 1 = At place 1<br>☐ 4 = I didn't get help	☐ 2 = At place 2	☐ 3 = At place 3

Do Not Circulate

Today's Date					
d	d	m	m	y	y

Study ID				

	Place 1	Place 2	Place 3
**e. If you traveled from home, how would you travel to that place?** MARK ALL THAT APPLY	❑ 1 = Walked<br>❑ 2 = Public transport<br>❑ 3 = Private taxi<br>❑ 4 = Friend/family lift<br>❑ 5 = Drove own car<br>❑ 6 = Other	❑ 1 = Walked<br>❑ 2 = Public transport<br>❑ 3 = Private taxi<br>❑ 4 = Friend/family lift<br>❑ 5 = Drove own car<br>❑ 6 = Other	❑ 1 = Walked<br>❑ 2 = Public transport<br>❑ 3 = Private taxi<br>❑ 4 = Friend/family lift<br>❑ 5 = Drove own car<br>❑ 6 = Other
**f. On average, how long would it take to travel to that place (one way) from the place where you stay?**	____ : ____<br>h h m m	____ : ____<br>h h m m	____ : ____<br>h h m m
**g. How much is the transport from the place where you stay to that place (one way)?**	ZAR ____ . ____	ZAR ____ . ____	ZAR ____ . ____
**h. How long did you spend at that place including waiting time, seeing the doctor/nurse, etc?**	____ : ____<br>h h m m	____ : ____<br>h h m m	____ : ____<br>h h m m
i. **How would you rate the quality of care that you had at the place(s) where you went for your Pap smear?** SHOW VISUAL AID	☐ 1 = Very good<br>☐ 2 = Good<br>☐ 3 = Neutral<br>☐ 4 = Fair<br>☐ 5 = Very bad	☐ 1 = Very good<br>☐ 2 = Good<br>☐ 3 = Neutral<br>☐ 4 = Fair<br>☐ 5 = Very bad	☐ 1 = Very good<br>☐ 2 = Good<br>☐ 3 = Neutral<br>☐ 4 = Fair<br>☐ 5 = Very bad
**j. Why is that?**	OPEN RESPONSE:	OPEN RESPONSE:	OPEN RESPONSE:

Do Not Circulate

Today's Date					
d	d	m	m	y	y

Study ID				

**k.** **Did you miss work because of your efforts to get a Pap smear? [**IF MORE THAN ONE PLACE: **This could be because of visits to any of the places you mentioned.]**<br><br>**l.** IF YES**, How many days?**	☐ 0= No → GO TO M<br>❑ 1 = Yes<br><br>______ days
m. **Did you lose any income from work or other sources because of your efforts to get a Pap smear?** [IF MORE THAN ONE PLACE: **This could be because of visits to any of the places you mentioned.**]<br><br>**n.** IF YES, **How much?**	☐ 0= No → GO TO O<br>☐ 1 = Yes<br><br>ZAR _________ . ________
**o.** **Were you unable to complete some of your daily tasks because of your efforts to get a Pap smear? [**IF MORE THAN ONE PLACE: **This could be because of visits to any of the places you mentioned.]**	☐ 0= No<br>☐ 1 = Yes
**I'd like to know about your expenses related to getting a Pap smear. [Considering all the places where you went,] did you have to pay for...**<br><br>p. **...medications?** IF YES, **How much?**<br><br>**q.** **... consultation(s) or doctor's fee(s)?** IF YES**, How much?**<br><br>**r.** **...child care while you were trying to get/getting the Pap smear?** IF YES, **How much?**	☐ 0= No<br>❑ 1 = Yes ZAR _____ . ______<br><br>☐ 0= No<br>❑ 1 = Yes ZAR _____ . ______<br><br>☐ 0= No<br>❑ 1 = Yes ZAR _____ . ______<br>❑ 2 = N/A (no children)

Do Not Circulate

Today's Date					
d	d	m	m	y	y

Study ID				

**s. ...<u>other</u> expenses related to getting your Pap smear?**<br><br>i. IF YES, **What kinds of things did you pay for?**<br><br>ii. AND IF YES, **How much did you pay for all of those expenses in total?**	☐ 0= No → GO TO T<br>❑ 1 = Yes<br><br>LIST: ____________________<br><br>ZAR _________ . ________
**t. Did anyone help you to pay for your expenses (e.g. transport, fees, etc)?**<br><br>u. IF YES, **how did they help?**	☐ 0= No → GO TO Q39<br>❑ 1 = Yes<br>❑ 2 = N/A (no expenses)<br><br>OPEN RESPONSE:

Do Not Circulate

BREAST CARE NEEDS AND COSTS

**39. a. Have you ever been screened for breast cancer or other breast related problems?**<br><br>b. If a=NO, **Why not?** PROVIDE RESPONSE AND THEN GO TO Q41.<br><br>c. IF YES, **When was the last time?** IF SHE HAS HAD MORE THAN ONE, ASK ABOUT THE LAST TIME.	❑ 0 = No<br>❑ 1 = Yes → GO TO Q39c<br><br>OPEN RESPONSE:<br><br>☐ 1 = In the last year<br>☐ 2 = More than one year ago → GO TO Q41

Today's Date					
d	d	m	m	y	y

Study ID				

**d. How many place(s) did you go to while trying to get screened?**	☐ 3 = Can't remember → GO TO Q41<br><br>☐ 1<br>☐ 2<br>☐ 3<br>☐ >3 COMPLETE Q40 FOR THE LAST THREE PLACES SHE WENT.

	Place 1	Place 2	Place 3
**40. a. When you were seeking your breast exam or help for your breast related problems, what kinds of place(s) did you go to?**	☐ 1 = Public clinic/hospital<br>☐ 2 = Private clinic/hospital<br>☐ 3 = Pharmacy/chemist<br>☐ 4 = Family/relative/friend<br>☐ 5 = Partner<br>☐ 6 =Traditional healer<br>☐ 7 = Other, _______	☐ 1 = Public clinic/hospital<br>☐ 2 = Private clinic/hospital<br>☐ 3 = Pharmacy/chemist<br>☐ 4 = Family/relative/friend<br>☐ 5 = Partner<br>☐ 6 =Traditional healer<br>☐ 7 = Other, _______	☐ 1 = Public clinic/hospital<br>☐ 2 = Private clinic/hospital<br>☐ 3 = Pharmacy/chemist<br>☐ 4 = Family/relative/friend<br>☐ 5 = Partner<br>☐ 6 =Traditional healer<br>☐ 7 = Other, _______
**b. Where was that place?**	NAME: ________________<br><br>CODE: ________________	NAME: ________________<br><br>CODE: ________________	NAME: ________________<br><br>CODE: ________________
**i. Was it close to home?**	☐ 0 = No ☐ 1 = Yes	☐ 0 = No ☐ 1 = Yes	☐ 0 = No ☐ 1 = Yes
**ii. Was it at or close to work?**	☐ 0 = No<br>☐ 1 = Yes<br>☐ 2 = N/A, not working	☐ 0 = No<br>☐ 1 = Yes<br>☐ 2 = N/A, not working	☐ 0 = No<br>☐ 1 = Yes<br>☐ 2 = N/A, not working
**c. How many times did you go to that place?**	______	______	______

Do Not Circulate

Today's Date					
d	d	m	m	y	y

Study ID				

	Place 1	Place 2	Place 3
**d. Where did you eventually get the help?** MARK ALL THAT APPLY	☐ 1 = At place 1<br>☐ 4 = I didn't get help	☐ 2 = At place 2	☐ 3 = At place 3
**e. If you traveled from home, how would you travel to that place?** MARK ALL THAT APPLY	❑ 1 = Walked<br>❑ 2 = Public transport<br>❑ 3 = Private taxi<br>❑ 4 = Friend/family lift<br>❑ 5 = Drove own car<br>❑ 6 = Other	❑ 1 = Walked<br>❑ 2 = Public transport<br>❑ 3 = Private taxi<br>❑ 4 = Friend/family lift<br>❑ 5 = Drove own car<br>❑ 6 = Other	❑ 1 = Walked<br>❑ 2 = Public transport<br>❑ 3 = Private taxi<br>❑ 4 = Friend/family lift<br>❑ 5 = Drove own car<br>❑ 6 = Other
**f. On average, how long would it take to travel to that place (one way) from the place where you stay?**	______ : ______<br>h h m m	______ : ______<br>h h m m	______ : ______<br>h h m m
**g. How much is the transport from the place where you stay to that place (one way)?**	ZAR ______ . _____	ZAR ______ . _____	ZAR ______ . _____
**h. How long did you spend at that place including waiting time, seeing the doctor/nurse, etc?**	______ : ______<br>h h m m	______ : ______<br>h h m m	______ : ______<br>h h m m

Do Not Circulate

Today's Date					
d	d	m	m	y	y

Study ID				

i. **How would you rate the quality of care that you had at the place(s) where you went for to get breast screening or care?** SHOW VISUAL AID<br><br>j. **Why is that?**	☐ 1 = Very good<br>☐ 2 = Good<br>☐ 3 = Neutral<br>☐ 4 = Fair<br>☐ 5 = Very bad<br><br>OPEN RESPONSE:	☐ 1 = Very good<br>☐ 2 = Good<br>☐ 3 = Neutral<br>☐ 4 = Fair<br>☐ 5 = Very bad<br><br>OPEN RESPONSE:	☐ 1 = Very good<br>☐ 2 = Good<br>☐ 3 = Neutral<br>☐ 4 = Fair<br>☐ 5 = Very bad<br><br>OPEN RESPONSE:

**k.** **Did you miss work because of your efforts to get breast screening or care? [**IF MORE THAN ONE PLACE: **This could be because of visits to any of the places you mentioned.]**<br><br>**l.** IF YES**, How many days?**	☐ 0= No → GO TO M<br>❑ 1 = Yes<br><br>_____ days
**m.** **Did you lose any income from work or other sources because of your efforts to get breast cancer screening or care? [**IF MORE THAN ONE PLACE: **This could be because of visits to any of the places you mentioned.]**<br><br>**n.** IF YES, **How much?**	☐ 0= No → GO TO O<br>☐ 1 = Yes<br><br>ZAR _________ . ________
**o.** **Were you unable to complete some of your daily tasks because of your efforts to get breast cancer screening or care? [**IF MORE THAN ONE PLACE: **This could be because of visits to any of the places you mentioned.]**	❑ 0= No<br>❑ 1 = Yes

Do Not Circulate

Today's Date					
d	d	m	m	y	y

Study ID				

**I'd like to know about your expenses related to your efforts to get breast cancer screening or care. [Considering all the places where you went,] did you have to pay for...**	
p. **...the medications?** IF YES, **How much?**	☐ 0= No<br>❑ 1 = Yes ZAR _____ . ______
**q. ... consultation(s) or doctor's fee(s)?** IF YES**, How much?**	☐ 0= No<br>❑ 1 = Yes ZAR _____ . ______
**r. ...child care while you were trying to get/getting the screening or care?** IF YES, **How much?**	☐ 0= No<br>❑ 1 = Yes ZAR _____ . ______<br>❑ 2 = N/A (no children)
**s. ...other expenses related to getting the breast cancer screening or care?**	☐ 0= No → GO TO T<br>❑ 1 = Yes
i. IF YES, **What kinds of things did you pay for?**	LIST: ____________________
ii. AND IF YES, **How much did you pay for all of those expenses in total?**	ZAR _________ . ________
**t. Did anyone help you to pay for your expenses (e.g. transport, fees, etc)?**	☐ 0= No → GO TO Q41<br>❑ 1 = Yes<br>❑ 2 = N/A (no expenses)
u. IF YES, **how did they help?**	OPEN RESPONSE:

Do Not Circulate

Today's Date					
d	d	m	m	y	y

Study ID				

SEXUALLY TRANSMITTED INFECTION NEEDS AND COSTS

**41. a. 1 Did you experience any symptoms that you think might be from a sexually transmitted infection, or "STI," in the past year?**	☐ 0 = No → GO TO Q43<br>☐ 1 = Yes<br>☐ 2 = Not sure/don't know → GO TO Q43
a. 2 IF YES, **What symptoms did you have?** TICK ALL THAT APPLY.	IF YES, INDICATE ALL THAT APPLY:<br>☐ 1 = Vaginal itching<br>☐ 2 = Pain and or burning when urinating<br>☐ 3 = Vaginal discharge with odour<br>☐ 4 = Lower back or abdominal pain<br>☐ 5 = Pain during sex<br>☐ 6 = Irregular bleeding between periods<br>☐ 7 = Other, specify ____________
**b.** IF a1=YES, **Did you seek help for those symptoms?**	☐ 0 = No<br>☐ 1 = Yes → GO TO Q41d
c. If b=NO, **Why not?** PROVIDE RESPONSE AND THEN GO TO Q43.	OPEN RESPONSE
**d. How many place(s) did you go to while trying to get care for your symptoms?**	☐ 1<br>☐ 2<br>☐ 3<br>☐ >3 COMPLETE Q42 FOR THE LAST THREE PLACES SHE WENT.

Do Not Circulate

Today's Date					
d	d	m	m	y	y

Study ID				

Do Not Circulate

	Place 1	Place 2	Place 3
**42. a. When you were seeking help for your STI symptoms, what kinds of place(s) did you go to?**	☐ 1 = Public clinic/hospital<br>☐ 2 = Private clinic/hospital<br>☐ 3 = Pharmacy/chemist<br>☐ 4 = Family/relative/friend<br>☐ 5 = Partner<br>☐ 6 =Traditional healer<br>☐ 7 = Other, _______	☐ 1 = Public clinic/hospital<br>☐ 2 = Private clinic/hospital<br>☐ 3 = Pharmacy/chemist<br>☐ 4 = Family/relative/friend<br>☐ 5 = Partner<br>☐ 6 =Traditional healer<br>☐ 7 = Other, _______	☐ 1 = Public clinic/hospital<br>☐ 2 = Private clinic/hospital<br>☐ 3 = Pharmacy/chemist<br>☐ 4 = Family/relative/friend<br>☐ 5 = Partner<br>☐ 6 =Traditional healer<br>☐ 7 = Other, _______
**b. Where was that place?**<br><br>**i. Was it close to home?**	NAME: _______________<br>CODE: _______________<br>☐ 0 = No ☐ 1 = Yes	NAME: _______________<br>CODE: _______________<br>☐ 0 = No ☐ 1 = Yes	NAME: _______________<br>CODE: _______________<br>☐ 0 = No ☐ 1 = Yes
**ii. Was it at or close to work?**	☐ 0 = No<br>☐ 1 = Yes<br>☐ 2 = N/A, not working	☐ 0 = No<br>☐ 1 = Yes<br>☐ 2 = N/A, not working	☐ 0 = No<br>☐ 1 = Yes<br>☐ 2 = N/A, not working
**c. How many times did you go to that place?**	______	______	______
**d. Where did you eventually get the help?** TICK ALL THAT APPLY	☐ 1 = At place 1<br><br>☐ 4 = I didn't get help	☐ 2 = At place 2	☐ 3 = At place 3
**e. If you traveled from home, how would you travel to that place?** MARK ALL THAT APPLY	❑ 1 = Walked<br>❑ 2 = Public transport<br>❑ 3 = Private taxi<br>❑ 4 = Friend/family lift<br>❑ 5 = Drove own car<br>❑ 6 = Other	❑ 1 = Walked<br>❑ 2 = Public transport<br>❑ 3 = Private taxi<br>❑ 4 = Friend/family lift<br>❑ 5 = Drove own car<br>❑ 6 = Other	❑ 1 = Walked<br>❑ 2 = Public transport<br>❑ 3 = Private taxi<br>❑ 4 = Friend/family lift<br>❑ 5 = Drove own car<br>❑ 6 = Other

Today's Date					
d	d	m	m	y	y

Study ID				

Do Not Circulate

	Place 1	Place 2	Place 3
**f. On average, how long would it take to travel to that place (one way) from the place where you stay?**	____ : ____ h h m m	____ : ____ h h m m	____ : ____ h h m m
**g. How much is the transport from the place where you stay to that place (one way)?**	ZAR ____ . ____	ZAR ____ . ____	ZAR ____ . ____
**h. How long did you spend at that place including waiting time, seeing the doctor/nurse, etc?**	____ : ____ h h m m	____ : ____ h h m m	____ : ____ h h m m
i. **How would you rate the quality of care that you had at the place where you got help for your menstrual problems?** SHOW VISUAL AID<br>**j. Why is that?**	☐ 1 = Very good<br>☐ 2 = Good<br>☐ 3 = Neutral<br>☐ 4 = Fair<br>☐ 5 = Very bad<br><br>OPEN RESPONSE:	☐ 1 = Very good<br>☐ 2 = Good<br>☐ 3 = Neutral<br>☐ 4 = Fair<br>☐ 5 = Very bad<br><br>OPEN RESPONSE:	☐ 1 = Very good<br>☐ 2 = Good<br>☐ 3 = Neutral<br>☐ 4 = Fair<br>☐ 5 = Very bad<br><br>OPEN RESPONSE:

**k. Did you miss work because of your efforts to get help for your STI symptoms? [**IF MORE THAN ONE PLACE: **This could be because of visits to any of the places you mentioned.]**	☐ 0= No → GO TO M<br>☐ 1 = Yes
**l.** IF YES**, How many days?**	______ days

Today's Date					
d	d	m	m	y	y

Study ID				

**m.** **Did you lose any income from work or other sources because of your efforts to get help for your STI symptoms? [**IF MORE THAN ONE PLACE: **This could be because of visits to any of the places you mentioned.]**	☐ 0= No → GO TO O<br>☐ 1 = Yes
**n.** IF YES, **How much?**	ZAR _________ . ________
**o.** **Were you unable to complete some of your daily tasks because of your efforts to get help for your STI symptoms? [**IF MORE THAN ONE PLACE: **This could be because of visits to any of the places you mentioned.]**	❑ 0= No<br>❑ 1 = Yes
**I'd like to know about your expenses related to getting help for your STI symptoms. [Considering all the places where you went] did you have to pay for...**	
p. **...<u>medications</u>?** IF YES, **How much?**	❑ 0= No<br>❑ 1 = Yes ZAR _____ . ______
**q.** **... <u>consultation</u>(s) or doctor's fee(s)?** IF YES**, How much?**	☐ 0= No<br>❑ 1 = Yes ZAR _____ . ______
**r.** **...<u>child care</u> while you were getting help for your STI symptoms?** IF YES, **How much?**	☐ 0= No<br>❑ 1 = Yes ZAR _____ . ______<br>❑ 2 = N/A (no children)
**s.** **...<u>other expenses</u> related to getting help for your STI symptoms?**	☐ 0= No → GO TO T<br>❑ 1 = Yes
i**.** IF YES, **What kinds of things did you pay for?**	LIST: ____________________
ii. AND IF YES, **How much did you pay for all of those expenses in total?**	ZAR _________ . ________

Do Not Circulate

Today's Date					
d	d	m	m	y	y

Study ID				

**t. Did anyone help you to pay for your expenses (e.g. transport, fees, etc)?**	☐ 0= No → GO TO Q43<br>☐ 1 = Yes<br>☐ 2 = N/A (no expenses)
u. IF YES, **how did they help?**	OPEN RESPONSE:

TOP NEEDS AND COSTS

Do Not Circulate

**In South Africa a woman has a right to end her pregnancy for certain reasons. I know that this is a sensitive issue for some people. I want to ask about your experiences with this. I will keep your answer confidential (along with all the information I am collecting today).**	
**43. a. Sometimes women have unplanned pregnancies that they do not want or cannot continue for lots of reasons. Did you have an unintended or unwanted pregnancy in last year?**	☐ 0 = No<br>☐ 1 = Yes<br>☐ 2 = Declined to answer
**b. Did you need to or decide that you wanted to have a termination of pregnancy, or TOP, at any time in the past year?**	☐ 0 = No → GO TO Q45<br>☐ 1 = Yes<br>☐ 2 = Declined to answer
**c.** IF b=YES, **Were you actually able to obtain a TOP in the past year?**	☐ 0 = No<br>☐ 1 = Yes → GO TO Q43e<br>☐ 2 = Declined to answer

**Today's Date**					
d	d	m	m	y	y

**Study ID**				

**d.** IF c=NO, **Why not?**	OPEN RESPONSE
**e.** IF c=YES, **How many place(s) did you go to while trying to get your TOP?**	☐ 1<br>☐ 2<br>☐ 3<br>☐ >3 COMPLETE Q44 FOR THE LAST THREE PLACES SHE WENT.

	**Place 1**	**Place 2**	**Place 3**
**44. a. When you were trying to get your TOP, what kinds of place(s) did you go to?**	☐ 1 = Public clinic/hospital<br>☐ 2 = Private clinic/hospital<br>☐ 3 = Pharmacy/chemist<br>☐ 4 = Family/relative/friend<br>☐ 5 = Partner<br>☐ 6 =Traditional healer<br>☐ 7 = Other, ________	☐ 1 = Public clinic/hospital<br>☐ 2 = Private clinic/hospital<br>☐ 3 = Pharmacy/chemist<br>☐ 4 = Family/relative/friend<br>☐ 5 = Partner<br>☐ 6 =Traditional healer<br>☐ 7 = Other, ________	☐ 1 = Public clinic/hospital<br>☐ 2 = Private clinic/hospital<br>☐ 3 = Pharmacy/chemist<br>☐ 4 = Family/relative/friend<br>☐ 5 = Partner<br>☐ 6 =Traditional healer<br>☐ 7 = Other, ________
**b. Where was that place?**	NAME: __________________<br>CODE: __________________	NAME: __________________<br>CODE: __________________	NAME: __________________<br>CODE: __________________
**i. Was it close to home?**	☐ 0 = No ☐ 1 = Yes	☐ 0 = No ☐ 1 = Yes	☐ 0 = No ☐ 1 = Yes
**ii. Was it at or close to work?**	☐ 0 = No<br>☐ 1 = Yes<br>☐ 2 = N/A, not working	☐ 0 = No<br>☐ 1 = Yes<br>☐ 2 = N/A, not working	☐ 0 = No<br>☐ 1 = Yes<br>☐ 2 = N/A, not working

Do Not Circulate

Today's Date					
d	d	m	m	y	y

Study ID				

**c. How many times did you go to that place?**	______	______	______
**d. Where did you eventually get the help?** MARK ALL THAT APPLY	☐ 1 = At place 1<br>☐ 4 = I didn't get help	☐ 2 = At place 2	☐ 3 = At place 3
**e. If you traveled from home, how would you travel to that place?** MARK ALL THAT APPLY	❑ 1 = Walked<br>❑ 2 = Public transport<br>❑ 3 = Private taxi<br>❑ 4 = Friend/family lift<br>❑ 5 = Drove own car<br>❑ 6 = Other	❑ 1 = Walked<br>❑ 2 = Public transport<br>❑ 3 = Private taxi<br>❑ 4 = Friend/family lift<br>❑ 5 = Drove own car<br>❑ 6 = Other	❑ 1 = Walked<br>❑ 2 = Public transport<br>❑ 3 = Private taxi<br>❑ 4 = Friend/family lift<br>❑ 5 = Drove own car<br>❑ 6 = Other
**f. On average, how long would it take to travel to that place (one way) from the place where you stay?**	______ : ______<br>h h m m	______ : ______<br>h h m m	______ : ______<br>h h m m
**g. How much is the transport from the place where you stay to that place (one way)?**	ZAR ______ . _____	ZAR ______ . _____	ZAR ______ . _____
**h. How long did you spend at that place including waiting time, getting the TOP, etc?**	______ : ______<br>h h m m	______ : ______<br>h h m m	______ : ______<br>h h m m
i. **How would you rate the quality of care that you had at the place(s) where you went for your TOP?** SHOW VISUAL AID	☐ 1 = Very good<br>☐ 2 = Good<br>☐ 3 = Neutral<br>☐ 4 = Fair<br>☐ 5 = Very bad	☐ 1 = Very good<br>☐ 2 = Good<br>☐ 3 = Neutral<br>☐ 4 = Fair<br>☐ 5 = Very bad	☐ 1 = Very good<br>☐ 2 = Good<br>☐ 3 = Neutral<br>☐ 4 = Fair<br>☐ 5 = Very bad

Do Not Circulate

Today's Date					
d	d	m	m	y	y

Study ID				

**j. Why is that?**	OPEN RESPONSE:	OPEN RESPONSE:	OPEN RESPONSE:

**k. Did you miss work because you were trying to get/getting a TOP? [**IF MORE THAN ONE PLACE: **This could be because of visits to any of the places you mentioned.]**	☐ 0= No → GO TO M<br>❑ 1 = Yes
**l.** IF YES**, How many days?**	______ days
**m. Did you lose any income from work or other sources while you were trying to get/getting your TOP? [**IF MORE THAN ONE PLACE: **This could be because of visits to any of the places you mentioned.]**	☐ 0= No → GO TO O<br>☐ 1 = Yes
**n.** IF YES, **How much?**	ZAR ________ . _______
**o. Were you unable to complete some of your daily tasks because you were trying to get/getting your TOP? [**IF MORE THAN ONE PLACE: **This could be because of visits to any of the places you mentioned.]**	❑ 0= No<br>❑ 1 = Yes
**I'd like to know about your expenses related to getting your TOP. [Considering all the places where you went,] did you have to pay for...**	
p. **...<u>medications</u>?** IF YES, **How much?**	☐ 0= No<br>❑ 1 = Yes ZAR _____ . ______
**q. ... <u>consultation</u>(s) or doctor's fee(s)?** IF YES**, How much?**	☐ 0= No<br>❑ 1 = Yes ZAR _____ . ______
**r. ...<u>child care</u> while you were getting the TOP?** IF YES, **How much?**	☐ 0= No<br>❑ 1 = Yes ZAR _____ . ______<br>❑ 2 = N/A (no childcare)

Do Not Circulate

Today's Date					
d	d	m	m	y	y

Study ID				

**s. ...<u>other expenses</u> related to getting the TOP?**<br><br>**i.** IF YES, **What kinds of things did you pay for?**<br><br>ii. AND IF YES, **How much did you pay for all of those expenses in total?**	☐ 0= No → GO TO T<br>❑ 1 = Yes<br><br>LIST: ____________________<br><br>ZAR _________ . ________
**t. Did anyone help you to pay for your expenses (e.g. transport, fees, etc)?**<br><br>u. IF YES, **how did they help?**	☐ 0= No → GO TO Q45<br>❑ 1 = Yes<br>❑ 2 = N/A (no expenses)<br><br>OPEN RESPONSE:

Do Not Circulate

GENDER BASED VIOLENCE NEEDS AND COSTS

**Violence against women is a common problem in South Africa. I'd like to ask you some questions about your experience with this kind of violence. I'd like to know about your relationship with your main partner. This is another sensitive issue. If you are uncomfortable talking about this, I understand. I can also refer you to someone for help, if you need that.**	
**45. a. Did you experience any kind of violence from your main partner during the past year? This could mean that your partner kicked, hit or punched you, that your partner made you feel scared or threatened, or that your partner forced or pressured you to have sex when you did not want to at any time during the past year?**	☐ 0 = No → GO TO Q47<br>☐ 1 = Yes<br>☐ 2 = Declined to answer → GO TO Q47

Today's Date					
d	d	m	m	y	y

Study ID				

**b.** IF a=YES, **Did you seek help for any of those problems?**	☐ 0 = No<br>☐ 1 = Yes → GO TO Q45d<br>☐ 2 = Declined to answer
c. If b=NO, **Why not?** PROVIDE RESPONSE AND THEN GO TO Q47.	OPEN RESPONSE
**d.** IF b=YES, **How many place(s) did you go to while trying to get help?**	☐ 1<br>☐ 2<br>☐ 3<br>☐ >3 COMPLETE Q46 FOR THE LAST THREE PLACES SHE WENT.

Do Not Circulate

	**Place 1**	**Place 2**	**Place 3**
**46. a. When you were seeking help for your experiences with gender based violence, what kinds of place(s) did you go to?**	☐ 1 = Public clinic/hospital<br>☐ 2 = Private clinic/hospital<br>☐ 3 = Pharmacy/chemist<br>☐ 4 = Family/relative/friend<br>☐ 5 = Partner<br>☐ 6 =Traditional healer<br>☐ 7 = Other, ________<br>☐ 8 = Police station<br>☐ 9 = Legal services provider<br>☐ 10 = Counselling service	☐ 1 = Public clinic/hospital<br>☐ 2 = Private clinic/hospital<br>☐ 3 = Pharmacy/chemist<br>☐ 4 = Family/relative/friend<br>☐ 5 = Partner<br>☐ 6 =Traditional healer<br>☐ 7 = Other, ________<br>☐ 8 = Police station<br>☐ 9 = Legal services provider<br>☐ 10 = Counselling service	☐ 1 = Public clinic/hospital<br>☐ 2 = Private clinic/hospital<br>☐ 3 = Pharmacy/chemist<br>☐ 4 = Family/relative/friend<br>☐ 5 = Partner<br>☐ 6 =Traditional healer<br>☐ 7 = Other, ________<br>☐ 8 = Police station<br>☐ 9 = Legal services provider<br>☐ 10 = Counselling service

Today's Date					
d	d	m	m	y	y

Study ID				

Do Not Circulate

**b. Where was that place?**	NAME: __________________<br>CODE: __________________	NAME: __________________<br>CODE: __________________	NAME: __________________<br>CODE: __________________
**i. Was it close to home?**	☐ 0 = No ☐ 1 = Yes	☐ 0 = No ☐ 1 = Yes	☐ 0 = No ☐ 1 = Yes
**ii. Was it at or close to work?**	☐ 0 = No<br>☐ 1 = Yes<br>☐ 2 = N/A, not working	☐ 0 = No<br>☐ 1 = Yes<br>☐ 2 = N/A, not working	☐ 0 = No<br>☐ 1 = Yes<br>☐ 2 = N/A, not working
**c. How many times did you go to that place?**	______	______	______
**d. Where did you eventually get the help?** TICK ALL THAT APPLY	☐ 1 = At place 1<br>☐ 4 = I didn't get help	☐ 2 = At place 2	☐ 3 = At place 3
**e. If you traveled from home, how would you travel to that place?** MARK ALL THAT APPLY	❑ 1 = Walked<br>❑ 2 = Public transport<br>❑ 3 = Private taxi<br>❑ 4 = Friend/family lift<br>❑ 5 = Drove own car<br>❑ 6 = Other	❑ 1 = Walked<br>❑ 2 = Public transport<br>❑ 3 = Private taxi<br>❑ 4 = Friend/family lift<br>❑ 5 = Drove own car<br>❑ 6 = Other	❑ 1 = Walked<br>❑ 2 = Public transport<br>❑ 3 = Private taxi<br>❑ 4 = Friend/family lift<br>❑ 5 = Drove own car<br>❑ 6 = Other
**f. On average, how long would it take to travel to that place (one way) from the place where you stay?**	______ : ______<br>h h m m	______ : ______<br>h h m m	______ : ______<br>h h m m

Today's Date					
d	d	m	m	y	y

Study ID				

**g. How much is the transport from the place where you stay to that place (one way)?**	ZAR ______ . _____	ZAR ______ . _____	ZAR ______ . _____
**h. How long did you spend at that place including waiting time, getting help, etc?**	_______ : _______<br>h h m m	_______ : _______<br>h h m m	_______ : _______<br>h h m m
i. **How would you rate the quality of care that you had at the place where you got help for your menstrual problems?** SHOW VISUAL AID<br><br>**j. Why is that?**	☐ 1 = Very good<br>☐ 2 = Good<br>☐ 3 = Neutral<br>☐ 4 = Fair<br>☐ 5 = Very bad<br><br>OPEN RESPONSE:	☐ 1 = Very good<br>☐ 2 = Good<br>☐ 3 = Neutral<br>☐ 4 = Fair<br>☐ 5 = Very bad<br><br>OPEN RESPONSE:	☐ 1 = Very good<br>☐ 2 = Good<br>☐ 3 = Neutral<br>☐ 4 = Fair<br>☐ 5 = Very bad<br><br>OPEN RESPONSE:

**k. Did you miss work because of your efforts to get help for your experiences with gender based violence? [**IF MORE THAN ONE PLACE: **This could be because of visits to any of the places you mentioned.]**	☐ 0= No → GO TO M<br>☐ 1 = Yes
**l.** IF YES**, How many days?**	______ days

Do Not Circulate

Today's Date					
d	d	m	m	y	y

Study ID				

**m. Did you lose any income from work or other sources because of your efforts to get help for your experiences with gender based violence? [**IF MORE THAN ONE PLACE: **This could be because of visits to any of the places you mentioned.]**<br><br>**n.** IF l=YES, **How much?**	☐ 0= No → GO TO O<br>☐ 1 = Yes<br><br>ZAR _________ . ________
**o. Were you unable to complete some of your daily tasks because of your efforts to get help for your experiences with gender based violence? [**IF MORE THAN ONE PLACE: **This could be because of visits to any of the places you mentioned.]**	❑ 0= No<br>❑ 1 = Yes
**I'd like to know about your expenses related getting help for your experiences with gender based violence. [Considering all the places where you went,] did you have to pay for...**<br><br>p. **...<u>medications</u>?** IF YES, **How much?**<br><br>**q. ... <u>consultation</u>(s) or doctor's fee(s)?** IF YES**, How much?**<br><br>**r. ...<u>child care</u> while you were getting help for your experiences with gender based violence?** IF YES, **How much?**<br><br>**s. ...<u>other expenses</u> related to getting help for your experiences with gender based violence?**	 <br><br>❑ 0= No<br>❑ 1 = Yes ZAR _____ . ______<br><br>☐ 0= No<br>❑ 1 = Yes ZAR _____ . ______<br><br>☐ 0= No<br>❑ 1 = Yes ZAR _____ . ______<br>❑ 2 = N/A (no children)<br><br>☐ 0= No → GO TO T<br>❑ 1 = Yes

Do Not Circulate

Today's Date					
d	d	m	m	y	y

Study ID				

i. IF YES, **What kinds of things did you pay for?**<br><br>ii. AND IF YES, **How much did you pay for all of those expenses in total?**	LIST: ____________________<br><br>ZAR _________ . ________
**t. Did anyone help you to pay for your expenses (e.g. transport, fees, etc)?**<br><br>u. IF YES, **how did they help?**	☐ 0= No → GO TO Q47<br>❑ 1 = Yes<br>❑ 2 = N/A (no expenses)<br><br>OPEN RESPONSE:

Do Not Circulate

Today's Date					
d	d	m	m	y	y

Study ID				

Part D: Service Quality / Integration

Do Not Circulate

**We're almost done. I have a few more questions about how you access health care, and where you would like to get different kinds of services. You can see from my questions that there are lots of different kinds of women's health services.**			
**47. Earlier you said you have some health problems or conditions. How many health facilities do you go to regularly to take care of those health conditions?**<br>CHECK RESPONSE AGAINST CONDITIONS REPORTED IN Q17. IF MORE THAN 3 PLACES, ASK FOR PLACES VISITED MOST FREQUENTLY.	❑ 0 = Doesn't regularly go to any health facility for chronic conditions → GO TO Q50<br>❑ 1 = 1 place<br>❑ 2 = 2 places<br>❑ 3 = 3 places<br>❑ 4 = > 3 places<br>❑ 5 = N/A (doesn't have any chronic conditions) → GO TO Q50		
	**Place 1**	**Place 2**	**Place 3**
**48. What is the name and location of that facility / place?**	NAME: ______________<br>CODE: ______________	NAME: ______________<br>CODE: ______________	NAME: ______________<br>CODE: ______________
**49. a. Are you currently able to get contraception at that place?**	❑ 0 = No<br>❑ 1 = Yes → GO TO Q49c<br>❑ 2 = Don't know	❑ 0 = No<br>❑ 1 = Yes → GO TO Q49c<br>❑ 2 = Don't know	❑ 0 = No<br>❑ 1 = Yes → GO TO Q49c<br>❑ 2 = Don't know
b. IF 49a=NO OR DON'T KNOW, **Would you like to be able to get contraception there?**	❑ 0 = No<br>❑ 1 = Yes<br>❑ 2 = Don't know	❑ 0 = No<br>❑ 1 = Yes<br>❑ 2 = Don't know	❑ 0 = No<br>❑ 1 = Yes<br>❑ 2 = Don't know
**c. Can you get other women's health services there?**	❑ 0 = No<br>❑ 1 = Yes<br>❑ 2 = Don't know	❑ 0 = No<br>❑ 1 = Yes<br>❑ 2 = Don't know	❑ 0 = No<br>❑ 1 = Yes<br>❑ 2 = Don't know

Today's Date					
d	d	m	m	y	y

Study ID				

SKIP Q50 IF SHE DOESN'T HAVE ANY CHILDREN.<br><br>**50. a. Can you currently get women's health services in the places where you get health services for your children?**<br><br>**b. Would you like to be able to get women's health services in the places where you get health services for your children?**<br><br>**c. Why is that?**	☐ 0= No<br>❑ 1 = Yes → GO TO Q51<br><br>☐ 0= No<br>❑ 1 = Yes<br><br>OPEN RESPONSE:
**51. a. When you visit the doctor or nurse for other health conditions or problems, would you like him or her to <u>spontaneously</u> give you information about women's health issues – like when to get screening for cervical cancer, breast cancer or information about contraceptive methods?**<br><br>**b. Why is that?**	☐ 0= No<br>❑ 1 = Yes<br><br>OPEN RESPONSE:
**52. We would like to follow up with some of the women who responded to this survey to talk to them more about women's health services and what they think could be done to improve services in public clinics and hospitals. If you are selected, could we contact you to ask if you would like to participate in another interview? It would be at a different location.**	☐ 0 = No<br>☐ 1 = Yes<br><br>IF YES, ENTER CONTACT INFORMATION ON THE CONTACT PAGE.

Do Not Circulate

Today's Date					
d	d	m	m	y	y

Study ID				

Interviewer initials and date:	
Interviewer comments:	

Do Not Circulate

Today's Date					
d	d	m	m	y	y

Study ID				

IF COMPLETED, REMOVE THIS PAGE AND STORE WITH THE CONSENT FORMS.

CONTACT INFORMATION

PHONE NUMBER 1:

PHONE NUMBER 2:

PHONE NUMBER 3:

Do Not Circulate
